# Efficient "Shotgun" Inference of Neural Connectivity from Highly Sub-sampled Activity Data

**DOI:** 10.1371/journal.pcbi.1004464

**Published:** 2015-10-14

**Authors:** Daniel Soudry, Suraj Keshri, Patrick Stinson, Min-hwan Oh, Garud Iyengar, Liam Paninski

**Affiliations:** 1 Department of Statistics, Department of Neuroscience, the Center for Theoretical Neuroscience, the Grossman Center for the Statistics of Mind, the Kavli Institute for Brain Science, and the NeuroTechnology Center, Columbia University, New York, New York, United States of America; 2 Department of Industrial Engineering and Operations Research, Columbia University, New York, New York, United States of America; University of Tübingen and Max Planck Institute for Biologial Cybernetics, GERMANY

## Abstract

Inferring connectivity in neuronal networks remains a key challenge in statistical neuroscience. The “common input” problem presents a major roadblock: it is difficult to reliably distinguish causal connections between pairs of observed neurons versus correlations induced by common input from unobserved neurons. Available techniques allow us to simultaneously record, with sufficient temporal resolution, only a small fraction of the network. Consequently, naive connectivity estimators that neglect these common input effects are highly biased. This work proposes a “shotgun” experimental design, in which we observe multiple sub-networks briefly, in a serial manner. Thus, while the full network cannot be observed simultaneously at any given time, we may be able to observe much larger subsets of the network over the course of the entire experiment, thus ameliorating the common input problem. Using a generalized linear model for a spiking recurrent neural network, we develop a scalable approximate expected loglikelihood-based Bayesian method to perform network inference given this type of data, in which only a small fraction of the network is observed in each time bin. We demonstrate in simulation that the shotgun experimental design can eliminate the biases induced by common input effects. Networks with thousands of neurons, in which only a small fraction of the neurons is observed in each time bin, can be quickly and accurately estimated, achieving orders of magnitude speed up over previous approaches.

## Author Summary

Optical imaging of the activity in a neuronal network is limited by the scanning speed of the imaging device. Therefore, typically, only a small fixed part of the network is observed during the entire experiment. However, in such an experiment, it can be hard to infer from the observed activity patterns whether (1) a neuron A directly affects neuron B, or (2) another, unobserved neuron C affects both A and B. To deal with this issue, we propose a “shotgun” observation scheme, in which, at each time point, we observe a small changing subset of the neurons from the network. Consequently, many fewer neurons remain completely unobserved during the entire experiment, enabling us to eventually distinguish between cases (1) and (2) given sufficiently long experiments. Since previous inference algorithms cannot efficiently handle so many missing observations, we develop a scalable algorithm for data acquired using the shotgun observation scheme, in which only a small fraction of the neurons are observed in each time bin. Using this kind of simulated data, we show the algorithm is able to quickly infer connectivity in spiking recurrent networks with thousands of neurons.

This is a *PLOS Computational Biology Methods* paper

## Introduction

It is now possible to image hundreds of neurons simultaneously at high spatiotemporal resolution [1] or tens of thousands of neurons at low spatiotemporal resolution [2]. The number of recorded neurons is expected to continue to grow exponentially [3]. This, in principle, provides the opportunity to infer the “functional” (or “effective”) connectivity of neuronal networks, *i.e*. a statistical estimate of how neurons are affected by each other, and by a stimulus. The ability to accurately estimate large, possibly time-varying, neural connectivity diagrams would open up an exciting new range of fundamental research questions in systems and computational neuroscience [4]. Therefore, the task of estimating connectivity from neural activity can be considered one of the central problems in statistical neuroscience.

Naturally, such a central problem has attracted much attention in recent years (see section 8). Perhaps the biggest challenge here involves the proper accounting for the activity of unobserved neurons. Despite rapid progress in simultaneously recording activity in massive populations of neurons, it is still beyond the reach of current technology to simultaneously monitor a complete large network of spiking neurons at high temporal resolution. Since connectivity estimation relies on the analysis of the the activity of neurons in relation to their inputs, the inability to monitor all of these inputs can result in persistent errors in the connectivity estimation due to model miss-specification. More specifically, “common input” errors, in which correlations due to shared inputs from unobserved neurons are mistaken for direct, causal connections, plague most naive approaches to connectivity estimation. Developing a robust approach for incorporating the latent effects of such unobserved neurons remains an area of active research in connectivity analysis (see section 8).

In this paper we propose an experimental design which can greatly ameliorate these common-input problems. The idea is simple: if we cannot observe all neurons in a network simultaneously, perhaps we can instead observe many overlapping sub-networks in a serial manner over the course of a long experiment. Then we can use statistical techniques to patch the full estimated network back together, analogous to “shotgun” genetic sequencing [5]. Obviously, it is not feasible to purposefully sample from many distinct sub-networks at many different overlapping locations using multi-electrode recording arrays, since multiple re-insertions of the array would lead to tissue damage. However, fluorescence-based imaging of neuronal calcium [6, 7] (or, perhaps in the not-too-distant future, voltage [8]) makes this approach experimentally feasible.

For example, such a shotgun approach could be highly beneficial and relatively straightforward to implement using a 3D acousto-optical deflector microscope [1]. Using such a microscope, one can scan a volume of 400 × 400 × 500 *μm*, which contains approximately 8000 cells. In normal use, the microscope’s 50kHz sampling rate allows for a frame rate of about 6Hz when scanning the entire volume. Unfortunately, this frame rate is too low for obtaining reliable connectivity estimates, which requires a frame rate of at least 30Hz [9]. However, we can increase the effective frame rate to 30Hz by using a shotgun approach. We simply divide the experimental duration into segments, where in each segment we scan only 20% of the network. As a side benefit of this shotgun approach, photobleaching and phototoxicity (two of the most important limitations on the duration of these experiments [10]) are reduced, since only a subset of the network is illuminated and imaged at any given time.

Connectivity estimation with missing observations is particularly challenging (section 9). Fortunately, as we show here, given the shotgun sampling scheme, we do not have to infer the unobserved spikes. We considerably simplify the network model loglikelihood using the expected loglikelihood approximation [11–13], and a generalized Central Limit Theorem (CLT) [14] argument to approximate the neuronal input as a Gaussian variable when the size of the network is large. This approximate loglikelihood and its gradients depend only on the empiric second order statistics of the spiking process (mean spike rate and spike correlations). Importantly, these approximate sufficient statistics can be calculated, even with partial observations, by simply “ignoring” any unobserved activity (section 3.6).

In order to obtain an accurate estimation of the connectivity, posterior distributions involving this simplified loglikelihood (along with various types of prior information about network connectivity) can be efficiently maximized. Using a sparsity inducing prior on the weights, we demonstrate numerically the effectiveness of our approach on simulated recurrent networks of spiking neurons. First, we demonstrate that the shotgun experimental design can largely eliminate the biases induced by common input effects (section 4). Then, we show that we can quickly infer connectivity for large networks, with a low fraction of neurons observed in each time bin (section 5). For example, our algorithm can be used to infer the connectivity of a sparse network with *O*(10^3^) neurons and *O*(10^5^) connections, given *O*(10^6^) time bins of spike data in which only 10% − 20% of the neurons are observed in each time bin. On a standard laptop, simulating such a network takes about half an hour, while inference takes a few minutes. This is faster than previous approaches by orders of magnitude, even when all spikes are observed (section 6.2). Our parameter scans suggest that our method is robust, and could be used for arbitrarily low observation ratios and an arbitrarily large number of neurons, given long enough experiments. We will discuss the outlook for experimental realizations of the proposed approach below, after presenting the basic methodology and simulated results. The supplementary material S1 Text contains the full details of the mathematical derivations and the numerical simulations.

## Methods

### 1 Preliminaries

#### 1.1 General Notation

A boldfaced letter **x** denotes a vector with components *x*_*i*_, a boldfaced capital letter **X** denotes a matrix with components *X*_*i*, *j*_, **X** ^(*k*)^ denotes the *k*-th matrix in a list, and **X**_⋅, *k*_ (**X**_*k*, ⋅_) the *k*-th column (row) vector of matrix **X**. For **X** ∈ ℝ^*N*×*T*^ we define the empiric average and variance

$$\begin{array}{ccc} {\left\langle X_{i,t}\right\rangle }_{T} & ≜ & \frac{1}{T}\sum_{t=1}^{T}X_{i,t}\;\;;\;\;{\text{Var}}_{T}(X_{i,t})≜\frac{1}{T}\sum_{t=1}^{T}{\left(X_{i,t}-{\left\langle X_{i,t}\right\rangle }_{T}\right)}^{2} \end{array}$$

Note the above expressions do not depend on *t*, despite the *t* index, which is maintained for notational convenience. For any condition *A*, we make use of 𝓘{*A*}, the indicator function (𝓘{*A*} = 1 if *A* holds, and zero otherwise). We define *δ*_*i*, *j*_ ≜ 𝓘{*i* = *j*}, Kronecker’s delta function. If **x** ∼ 𝓝(**μ**,**Σ**), then **x** is Gaussian random vector with mean **μ** and covariance matrix **Σ**, and we denote its density by 𝓝(**x**∣**μ**,**Σ**).

#### 1.2 Model

We use a discrete-time neural network. The neurons, indexed from *i* = 1 to *N*, produce spikes in time bins indexed from *t* = 1 to *T*. The spiking matrix **S** is composed of variables *S*_*i*, *t*_ indicating the number of spikes neuron *i* produces at time bin *t*. We assume each neuron *i* generates spikes *S*_*i*, *t*_ ∈ {0,1} according to a Generalized Linear neuron Model (GLM [15–17]), with a logistic probability function

$$\begin{array}{c} P(S_{i,t}=1|U_{i,t})=f(U_{i,t})≜\frac{1}{1+e^{-U_{i,t}}}\;, \end{array}$$ (1)

depending on the the input *U*_*i*, *t*_ it receives from other neurons, as well as from some external stimulus. Such a logistic function is adequate if any time bin rarely contains more than one spike (this is approximately true if the time bin is much smaller than the average inter-spike interval). The input to all the neurons in the network is therefore

$$\begin{array}{c} U_{\cdot ,t}\;≜\;WS_{\cdot ,t-1}+b+GX_{\cdot ,t}\;, \end{array}$$ (2)

where *b*_*i*_ is the (unknown) bias of neuron *i*; **X** ∈ ℝ^*D*×*T*^ are the external inputs (with *D* being the number of inputs); **G** ∈ ℝ^*N*×*D*^ is the input gain; and **W** ∈ ℝ^*N*×*N*^ is the (unknown) network connectivity matrix. The diagonal elements *W*_*i*, *i*_ of the connectivity matrix correspond to the post spike filter accounting for the cell’s own post-spike effects (*e.g*., refractory period), while the off-diagonal terms *W*_*i*, *j*_ represent the connection weights from neuron *j* to neuron *i*. The bias *b*_*i*_ controls the mean spike probability (firing rate) of neuron *i*. The external input **X** can represent a direct (*e.g*., light activated ion channels) or sensory stimulation of neurons in the network. The input gain **G** is a spatial filter that acts on the input **X**. We assume that the initial spiking pattern is drawn from some fixed distribution *P*(**S**_⋅,0_).

To simplify notation, we have assumed in Eq 2 that **U**_⋅, *t*_ is only affected by spiking activity from the previous time bin (**W** **S**_⋅, *t*−1_). However, to include a longer history of the spiking activity, we can simply replace the vector **S**_⋅, *t*−1_ in Eq 2 with the concatenation of the vectors **S**_⋅, *t*−1_, …,**S**_⋅, *t*−*k*_ and obtain similar results.

#### 1.3 Task

Our goal is to infer the connectivity matrix **W**, biases **b** and the stimulus gain **G**. We assume that we have some prior information on the weights, and that we know *N*, and the external input **X**. We noiselessly observe a subset of the generated spikes. For simplicity we initially ignore the problem of inferring spikes from the experimental data, which requires spike sorting or deconvolution of fluorescence traces. Later, we will address this issue of spike inference numerically (see also [9, 18] for a more systematic analysis of this issue). We use a binary matrix **O** to indicate which neurons were observed, so

$$\begin{array}{c} O_{i,t}\;≜\;𝓘[S_{i,t}\;\text{was}\;\text{observed}]\;. \end{array}$$ (3)

Practically, if *O*_*i*, *t*_ = 1 neuron *i* was imaged for sufficiently long time and with a high enough frame rate around time bin *t* so that we can infer whether a spike occurred in time bin *t* with relative certainty.

### 2 Analytical results—Bayesian inference of the weights

We use a Bayesian approach to infer the unknown weights. Suppose initially, for simplicity, that all spikes are observed and that there is no external input (**G** = 0). In this case, the log-posterior of the weights, given the spiking activity, is

$$\begin{array}{c} \ln P(W|S,b)=\ln P(S|W,b)+\ln P_{0}(W)+C\;, \end{array}$$ (4)

where ln *P*(**S**∣**W**,**b**) is the loglikelihood, *P*_0_(**W**) is some prior on the weights (we do not assume a prior on the biases **b**), and *C* is some unimportant constant which does not depend on **W** or **b**. Our aim is to find the Maximum A Posteriori (MAP) estimator for **W**, together with the Maximum Likelihood (ML) estimator for **b**, by solving

$$\begin{array}{c} \max_{W,b}\ln P(W|S,b)\;. \end{array}$$ (5)

If **S** is fully observed, this problem can be straightforwardly optimized without requiring an approximation (though the optimization procedure can be slow). However, our goal is to provide an estimate when only a subset of **S** is observed. This cannot be easily done using standard method. To see this, we examine the likelihood of a GLM (recalling Eqs (1) and (2)),

$$\begin{array}{ccc} & & \ln P(S|W,b)\\ & = & \sum_{i=1}^{N}\sum_{t=1}^{T}\ln[\frac{e^{S_{i,t}U_{i,t}}}{1+e^{U_{i,t}}}] \end{array}$$ (6)

$$\begin{array}{c} =\sum_{i=1}^{N}\sum_{t=1}^{T}[S_{i,t}U_{i,t}-\ln(1+e^{U_{i,t}})]. \end{array}$$ (7)

This likelihood (Eq 7), and its gradients, both contain a sum over weighted spikes in *U*_*i*, *t*_ (the **WS**_⋅, *t*−1_ term in Eq 2), that cannot be evaluated if some spikes are missing, unless the missing spikes are accurately inferred (section E in S1 Text). However, methods for inferring these missing spikes are typically slow, and do not scale well.

To circumvent these issues, we will show the loglikelihood can be approximated with a simple form, under a few reasonable assumptions. Importantly, this simple form can be easily calculated even if there are missing observations (the full derivation is in section 2.1). Using an extension of the techniques in [11–13], we develop an approximation to the likelihood based on the law of large numbers (the “expected loglikelihood” approximation) together with a generalized Central Limit Theorem (CLT) argument [14], in which we approximate the neuronal input to be Gaussian near the limit *N* → ∞; then we calculate the “profile likelihood” max_**b**_ln *P*(**S**∣**W**,**b**), in which the bias term has been substituted for its maximizing value. The end result is

$$\begin{array}{ccc} \max_{b}\ln P(S|W,b) & \approx & T\sum_{i=1}^{N}[\sum_{j=1}^{N}[W_{i,j}\Sigma_{i,j}^{\left(1\right)}]-h(m_{i})\sqrt{1+\frac{\pi }{8}\sum_{k,j}W_{i,j}\Sigma_{k,j}^{\left(0\right)}W_{i,k}}]\;, \end{array}$$ (8)

where we defined the mean spike probability, spike covariance, and the entropy function, respectively:

$$\begin{array}{c} m_{i}\;≜\;{\left\langle S_{i,t}\right\rangle }_{T} \end{array}$$ (9)

$$\begin{array}{c} \Sigma_{i,j}^{\left(k\right)}\;≜\;{\left\langle S_{i,t}S_{j,t-k}\right\rangle }_{T}-m_{i}m_{j} \end{array}$$ (10)

$$\begin{array}{c} h(m_{i})\;≜\;-m_{i}\ln m_{i}-(1-m_{i})\ln(1-m_{i})\;. \end{array}$$ (11)

A few comments:

1. Importantly, the profile loglikelihood (Eq 8) depends only on the first and second order moments of the spikes **m** and **Σ** ^(*k*)^ for *k* ∈ {0,1}. When all of the neurons in the network are observed, these moments can be computed directly, and therefore the empirical moments are approximate sufficient statistics, whose value contains all the information needed to compute any estimate of ****W****. As we explain in section 3, these empirical moments can be estimated even if only a subset of the spikes is observed.
2. As we show in section A in S1 Text, the profile loglikelihood (Eq 8) is concave, so it is easy to maximize the log-posterior and obtain the MAP estimate of **W**. This can be done orders of magnitude faster than in the standard MAP estimate (section 6.2), since Eq 8 does not contain a sum over time, as the original loglikelihood (Eq 7). Moreover, the optimization problem of finding the MAP estimate can be parallelized over the rows of **W**.
  $$\begin{array}{c} \max_{b}\ln P(W|S,b)=\sum_{i}\max_{b}\ln P(W_{i,\cdot }|S,b), \end{array}$$ (12)
  because the profile loglikelihood (Eq 8) decomposes over the rows of **W**, as does the L1 prior we will use here (Eq 46).
3. As we show in section A in S1 Text, we can straightforwardly differentiate Eq 8 to analytically obtain the gradient, Hessian, and even the maximizer of this profile loglikelihood, which is the maximum likelihood estimate of **W**. However, due to the nature of the integral approximation we make in Eq 14, more accurate results are obtained if we first differentiate the original loglikelihood (Eq 7), and then use the expectation approximation (together with the generalized CLT argument). This results in an adjustment of the loglikelihood gradient (section D in S1 Text).
4. A novel aspect of this work is that we apply the Expected LogLikelihood (ELL) approximation to a GLM with a bounded logistic rate function (Eq 1), which allows us to infer connectivity in *recurrent* neural networks. In contrast, previous works that used the ELL approximation [11–13] focused on single neuron responses, with an emphasis on either a Poisson neuron model with an exponential rate function, or simpler linear Gaussian models. Such models are less suitable for recurrent neural networks. Exponential rate functions cause instability, as the activity tends to to diverge, unless both the weights and the time bins are small. Linear networks are not a very realistic model for a neural network, and do not perform well in inferring synaptic connectivity [19].
5. Though we assumed a logistic neuron model (Eq 1), similar results can be derived for any spiking neuron model for which 1−*f*(*x*) = *f*(−*x*). This is explained in section A.3 S1 Text.
6. Though we assumed the network does not have a stimulus (**G** = 0), one can be incorporated into the inference procedure. To do so, we treat the stimulus **X**_⋅, *t*_ simply as the activity of additional, fully observed, neurons (albeit *X*_*i*, *t*_ ∈ ℝ while *S*_*i*, *t*_ ∈ {0,1}). Specifically, we define a new “spikes” matrix **S** ^new^ ≜ (**S** ^⊤^,**X** ^⊤^)^⊤^, a new connectivity matrix
  $$\begin{array}{c} W^{\text{new}}\;≜\;(\begin{array}{cc} W & G\\ 0^{D\times N} & 0^{D\times D} \end{array})\;, \end{array}$$
  and a new observation matrix **O** ^new^ ≜ (**O** ^⊤^,1^*T*×*D*^)^⊤^. Repeating the derivations for **S** ^new^,**W** ^new^ and **O** ^new^, we obtain the same profile loglikelihood. Once it is used to infer **W** ^new^, we extract the estimates of **W** and **G** from their corresponding blocks in **W** ^new^.
7. For simplicity and efficiency, we chose to focus on MAP estimates. However, other types of estimators and Bayesian approaches (*e.g*., MCMC, variational Bayes) might be used with this approximate loglikelihood, and should be explored in future work.

#### 2.1 Derivation of the simplified loglikelihood (Eq 8)

Recall Eqs (1) and (2) with **G** = 0. Combining both for times *t* = 1, ⋯, *T* and neurons *i* = 1, …, *N*, we obtain

$$\begin{array}{ccc} & & \ln P(S|W,b)\\ & & \;=\sum_{i=1}^{N}\sum_{t=1}^{T}[S_{i,t}U_{i,t}-\ln(1+e^{U_{i,t}})],\\ & & \;=T\sum_{i=1}^{N}[{\left\langle S_{i,t}U_{i,t}\right\rangle }_{T}-{\left\langle \ln(1+e^{U_{i,t}})\right\rangle }_{T}]\\ & & \;\overset{\left(1\right)}{\approx }T\sum_{i=1}^{N}[{\left\langle S_{i,t}U_{i,t}\right\rangle }_{T}-\int \ln(1+e^{x})𝓝(x|{\left\langle U_{i,t}\right\rangle }_{T},{\text{Var}}_{T}(U_{i,t}))dx]\\ & & \;\overset{\left(2\right)}{\approx }T\sum_{i=1}^{N}[{\left\langle S_{i,t}U_{i,t}\right\rangle }_{T}-\sqrt{1+\pi {\text{Var}}_{T}(U_{i,t})/8}\ln(1+\exp(\frac{{\left\langle U_{i,t}\right\rangle }_{T}}{\sqrt{1+\pi {\text{Var}}_{T}(U_{i,t})/8}}))]\\ & & \;\overset{\left(3\right)}{=}T\sum_{i=1}^{N}\sum_{j=1}^{N}W_{i,j}\Sigma_{i,j}^{\left(1\right)}+m_{i}b_{i}\\ & & \;-\sqrt{1+\pi \sum_{k,j}W_{i,j}\Sigma_{k,j}^{\left(0\right)}W_{i,k}/8}\ln(1+\exp(\frac{\sum_{k=1}^{N}W_{i,k}m_{k}+b_{i}}{\sqrt{1+\pi \sum_{k,j}W_{i,j}\Sigma_{k,j}^{\left(0\right)}W_{i,k}/8}}))\;, \end{array}$$ (13)

where we used the following:

1. The neuronal input, as a sum of *N* variables, converges to a Gaussian distribution, in the limit of large *N*, under rather general conditions [14]. Formally, we need to make sure these are fulfilled for our approximate method to work, which can become even more challenging with the addition of (arbitrary) external inputs. However, such a generalized CLT-based approximation tends to work quite well even when the neuronal input is not strictly Gaussian [20–22]. This robustness is demonstrated numerically in our simulations.
2. The integral approximation
  $$\begin{array}{c} \int_{-\infty }^{\infty }\log(1+e^{x})𝓝(x|\mu ,\sigma^{2})dx\approx \sqrt{1+\pi \sigma^{2}/8}\log(1+\exp(\frac{\mu }{\sqrt{1+\pi \sigma^{2}/8}}))\;, \end{array}$$ (14)
  from Eq 8 from [23]. This approximation is valid on a limited range, and is inaccurate for low *μ*. However, this can be corrected by adjusting by the gradient, as we explain in section D S1 Text.
3. Eq 2 for the neuronal input and Eqs (9)–(10) for the spike statistics, which yields
  $$\begin{array}{ccc} {\left\langle U_{i,t}\right\rangle }_{T} & = & \sum_{k=1}^{N}W_{i,k}m_{k}+b_{i}\\ {\text{Var}}_{T}(U_{i,t}) & = & \sum_{k=1}^{N}\sum_{j=1}^{N}W_{i,j}\Sigma_{k,j}^{\left(0\right)}W_{i,k}\;. \end{array}$$

Though the loglikelihood in Eq 13 has already become tractable (and depends only on the sufficient statistics from Eqs (9)–(10), we can simplify it further by maximizing it over **b**. To do so, we equate the derivative of the simplified loglikelihood (Eq 13) to zero

$$\begin{array}{ccc} \frac{d}{db_{i}}\ln P(S|W,b) & = & 0\;. \end{array}$$

Solving this equation, we obtain

$$\begin{array}{c} b_{i}=\sqrt{1+\pi \sum_{k,j}W_{i,j}\Sigma_{k,j}^{\left(0\right)}W_{i,k}/8}\ln(\frac{m_{i}}{1-m_{i}})-\sum_{k=1}^{N}W_{i,k}m_{k}\;. \end{array}$$ (15)

Substituting this maximizer into Eq 13, we obtain Eq 8.

### 3 Observation schemes

**Fig 1:**
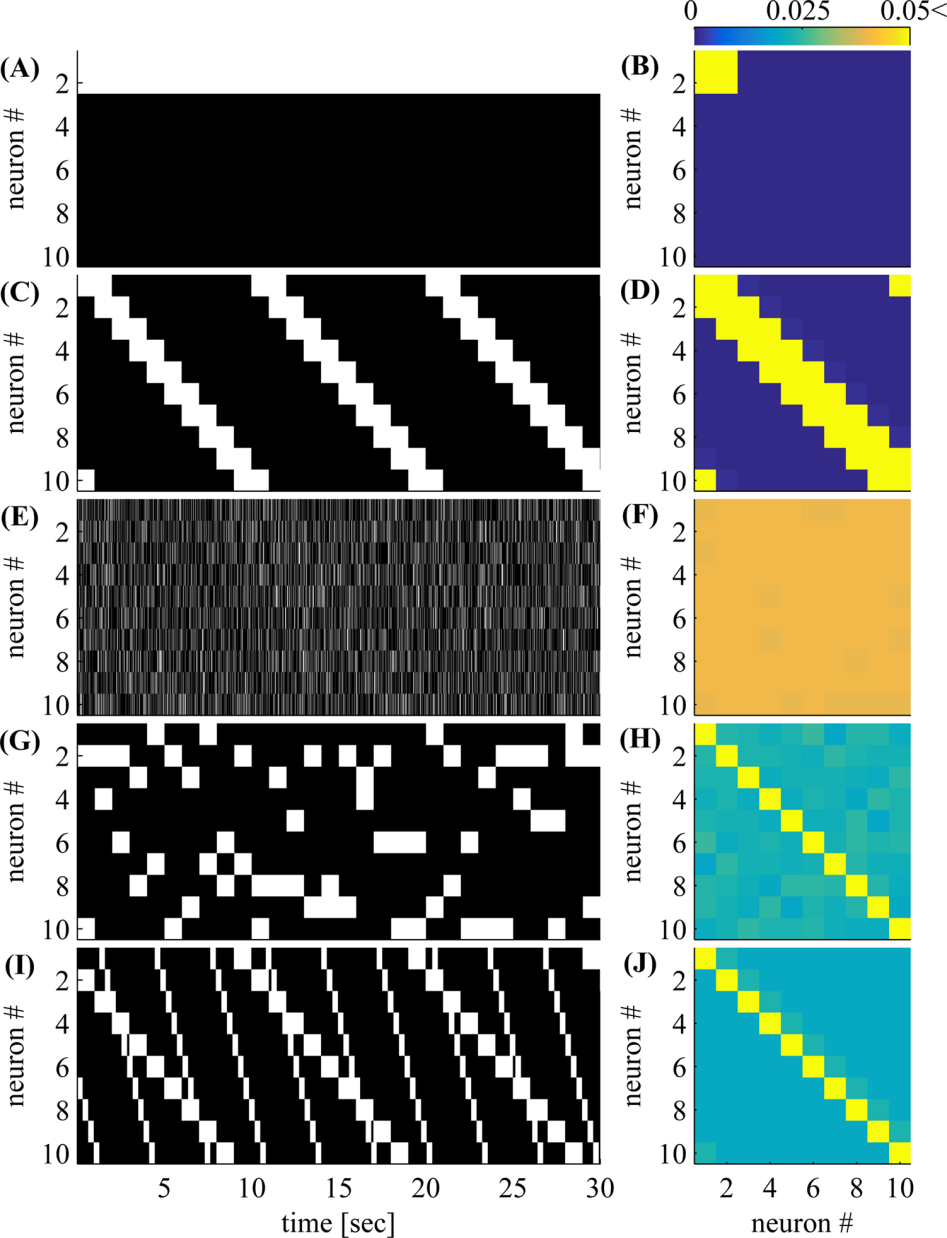
Observation scheme examples. In each scheme (the different rows) we observe two out of ten neurons in each time bin: **(A,B)** Fixed subset **(C,D)** Serial **(E,F)** Fully Random, **(G,H)** Random Blocks, and **(I,J)** double serial. *Left* (A,C,E,G,I): A sample of the observations **O** demonstrating the scanning method (a zero-one matrix, Eq 3). *Right* (B,D,F,H,J): empirical frequency of observed neuron pairs ${\left\langle O_{i,t}O_{j,t-1}\right\rangle }_{T}=\frac{1}{T}\sum_{t=1}^{T}O_{i,t}O_{j,t-1}$, with saturated colors to accentuate differences between methods (all values above 0.05 are shown in yellow). In the “fixed” scheme, some neurons are never observed. In the “serial” scheme some neuronal pairs are never observed. In all other schemes, all neuronal pairs are observed, so we can estimate the empirical moments using Eqs (16)–(17) and infer connectivity. In the two bottom schemes, observations are collected in persistent blocks, so neuron pairs which are close to the diagonal are observed more often.

As we showed in section 2, in order to infer network connectivity, we just need to estimate the first and second empiric spike statistics, defined in Eqs (9)–(10). These statistics cannot be calculated exactly if some observations are missing; in this case they must be estimated, as we discuss in section 3.6 below. First, though, it is useful to discuss a few concrete examples of the partial network observation schemes we are considering (Fig 1). We discuss the pros and cons of each scheme in terms of both inferential and experimental constraints.

#### 3.1 Fixed subset observations

The simplest and most commonly used observation scheme is simply to image a fixed subset of the network. To increase the size of the subset, we must image with a lower frame rate. However, since we observe only a subset of the neurons and neuronal pairs (Fig 1A, 1B), we can estimate only a subset of the mean spike probabilities **m** and spike covariances **Σ** ^(*k*)^. If we attempt to infer connectivity using only these incomplete empirical moments (*i.e*., by “pretending” the unobserved neurons do not exist), a persistent bias may be present in our estimate. This is due to the common input problem, as discussed in more depth in section 4 below.

#### 3.2 Serial subset observations

An alternative natural approach is to continuously shift the observed subset (Fig 1C, 1D): observe a given volume of tissue, then move the microscope (or specimen) to the left a bit, then repeat this procedure in a scanning fashion. However, under this approach some neuron pairs are still never observed. Specifically, as can be seen in Fig 1D, if the size of the scanning block is *k*, we do not observe neuron pairs for which ∣*i*−*j*∣ > *k* (*i.e*., ⟨*O*_*i*, *t*_ *O*_*j*, *t*−1_⟩_*T*_ = 0 for these pairs). Since we do not observe all pairs, it is not always possible to infer the spike covariances (**Σ** ^(*k*)^, Eq 10), which may be required for inferring connectivity.

#### 3.3 Fully randomized subsets

In order to accurately infer spike covariances, we examine a different observation method. If we randomly generate our observations (*O*_*i*, *t*_ = 1 with probability *p*_obs_, and otherwise *O*_*i*, *t*_ = 0), then all neuron pairs are uniformly observed (*i.e*., ${\left\langle O_{i,t}O_{j,t-1}\right\rangle }_{T}=p_{\text{obs}}^{2}$, ∀*i*, *j*); Fig 1E, 1F. In this case, it is easy to estimate both **m** and **Σ** ^(*k*)^, as explained in section 3.6 below. However, this observation scheme is experimentally infeasible, as we cannot infer spikes from fluorescence traces if neurons are observed for too short a time.

#### 3.4 Persistent block observations

To facilitate spike inference from fluorescence traces, we can randomly select a block of *p*_obs_ *N* neurons from the network, and observe this block for a sufficiently long time and with a sufficiently high frame rate, so that all spikes within the block can be inferred accurately (Fig 1G, 1H). Again, we can easily estimate both **m** and **Σ** ^(*k*)^ (section 3.6).

Randomly selecting blocks can be technically challenging. Within the field of view, this can be done using an acousto-optical deflector [24] or a spatial light modulator [25]. Enlarging the field of view of these methods remains an open experimental challenge, however.

Of course, non-random block scanning approaches are also possible. An alternative approach could be to employ light sheet methods [2, 26] and then slowly rotate the angle the light sheet forms as it passes through the specimen; as this angle changes, we will collect statistics involving groups of neurons in different planes. The estimation of **m** and **Σ** ^(*k*)^ would remain straightforward in this case.

#### 3.5 Double serial scanning

Finally, we note that combinations of the above schemes are possible. One such approach uses two simultaneous serial scans. For example, we can use a lexicographic scheme with two scanners–we first observe a fixed subset with one scanner, and, at the same time, serially observe the remaining blocks of the network with another scanner. Then, we move the first scanner to a different area, and perform another full scan with the second. We continue this way until we have completed a full scan of the network with the first scanner. Alternatively, we do not have to wait until the second scanner has finished a complete scan of the network. Instead, we can continuously scan with both scanners. If the scanning periods are incommensurate (*i.e*., their ratio is an irrational number) then eventually, we will observe all neuron pairs, as illustrated in Fig 1I, 1J. Such a dual-scanning scheme would of course pose some significant engineering challenges, but recent progress in imaging technology provides some hope that these challenges will be surmountable [27].

#### 3.6 Moment estimation

Next, we explain how the empirical moments can be estimated when there are missing observations. Perhaps the simplest estimate of these mean spike probabilities ignores any missing observations and just re-normalizes the empirical sums accordingly:

$$\begin{array}{c} {\tilde{m}}_{i}\;≜\;\frac{{\left\langle O_{i,t}S_{i,t}\right\rangle }_{T}}{{\left\langle O_{i,t}\right\rangle }_{T}}\;. \end{array}$$ (16)

This estimate is consistent (*i.e*., ${\tilde{m}}_{i}\to m_{i}$ when *T* → ∞), since

$$\begin{array}{ccc} \frac{{\left\langle O_{i,t}S_{i,t}\right\rangle }_{T}}{{\left\langle O_{i,t}\right\rangle }_{T}} & \overset{\left(1\right)}{\to } & \frac{{\left\langle O_{i,t}\right\rangle }_{T}{\left\langle S_{i,t}\right\rangle }_{T}}{{\left\langle O_{i,t}\right\rangle }_{T}}\overset{\left(2\right)}{\to }{\left\langle S_{i,t}\right\rangle }_{T}=m_{i} \end{array}$$

where we have assumed that

1. The observation process is uncorrelated with the spikes.
2. ⟨*O*_*i*, *t*_⟩_*T*_ converges to a strictly positive limit ∀*i*.

The first condition is typically the case in most experiments. The second condition implies we observe each neuron for a large number of time bins; importantly, this condition does *not* hold in the fixed subset observation scheme. Similarly,

$$\begin{array}{ccc} {\tilde{\Sigma }}_{i,j}^{\left(k\right)} & \;≜\; & \frac{{\left\langle O_{i,t}O_{j,t-k}S_{i,t}S_{j,t-k}\right\rangle }_{T}}{{\left\langle O_{i,t}O_{j,t-k}\right\rangle }_{T}}-{\tilde{m}}_{i}{\tilde{m}}_{j}\to \Sigma^{\left(k\right)}\;, \end{array}$$ (17)

if we additionally assume that

3. ⟨*O*_*i*, *t*_ *O*_*j*, *t*−*k*_⟩_*T*_ converges to a strictly positive limit ∀*i*, *j* and ∀*k* ∈ {0,1}.

This third condition implies that we observe each neuron pair (either at the same time, or delayed) for a large number of time bins (recall that this condition does not hold in the serial observation scheme).

This direct approach is simple and computationally quite cheap. However, it seems to ignore potentially useful information: if we could “fill in” the activity in the unobserved bins of the *S*_*i*, *t*_ matrix, we would increase our effective sample size and therefore estimate the required moments more accurately. We have experimented with a couple approximate Bayesian methods for filling in this missing information (as detailed in more depth in the section E S1 Text), and somewhat surprisingly have found that they do not greatly improve the estimation accuracy, while imposing significant computational cost. See section 6.1 below for further details.

## Results

Our goal in this section is to demonstrate numerically that connectivity can be inferred, efficiently and accurately, from highly sub-sampled spike data. Readers can find the basic notation used in this section in Table 1. In section 4, we give a qualitative demonstration that the shotgun approach can be used to significantly decrease the usual persistent bias resulting from common inputs. In section 5, we perform quantitative tests to show that our estimation method is effective and robust; we perform parameter scans for various network sizes, observation probabilities, firing rates and connection sparsities. In section 6, we show that our Expected LogLikelihood (ELL) based estimation method is efficient, both statistically and computationally. Finally, in section 7, we infer connectivity from fluorescence measurements.

**Table 1:** Basic notation.

*N*	Total number of neurons
*T*	Total number of time bins
*p*_obs_	Empiric observation probability—the mean fraction of neurons observed at each time bin
*p*_conn_	Network sparsity—the average probability that two neurons are directly connected
**S**	*N* × *T* matrix of spike activity
**W**	*N* × *N* matrix of synaptic connection weights
****U****	*N* × *T* matrix of neuronal inputs
**b**	*N*×1 vector of neuronal biases
**O**	*N* × *T* binary matrix denoting when neurons are observed
**m**	*N*×1 vector of mean spike probability (firing rates)
**Σ** ^(*k*)^	*N* × *N* matrix of mean spike covariances with lag *k*

### 4 The common input problem

In this section we use a toy network with *N* = 50 neurons to visualize the common input problem, and its suggested solution—the “shotgun” approach.

**Fig 2:**
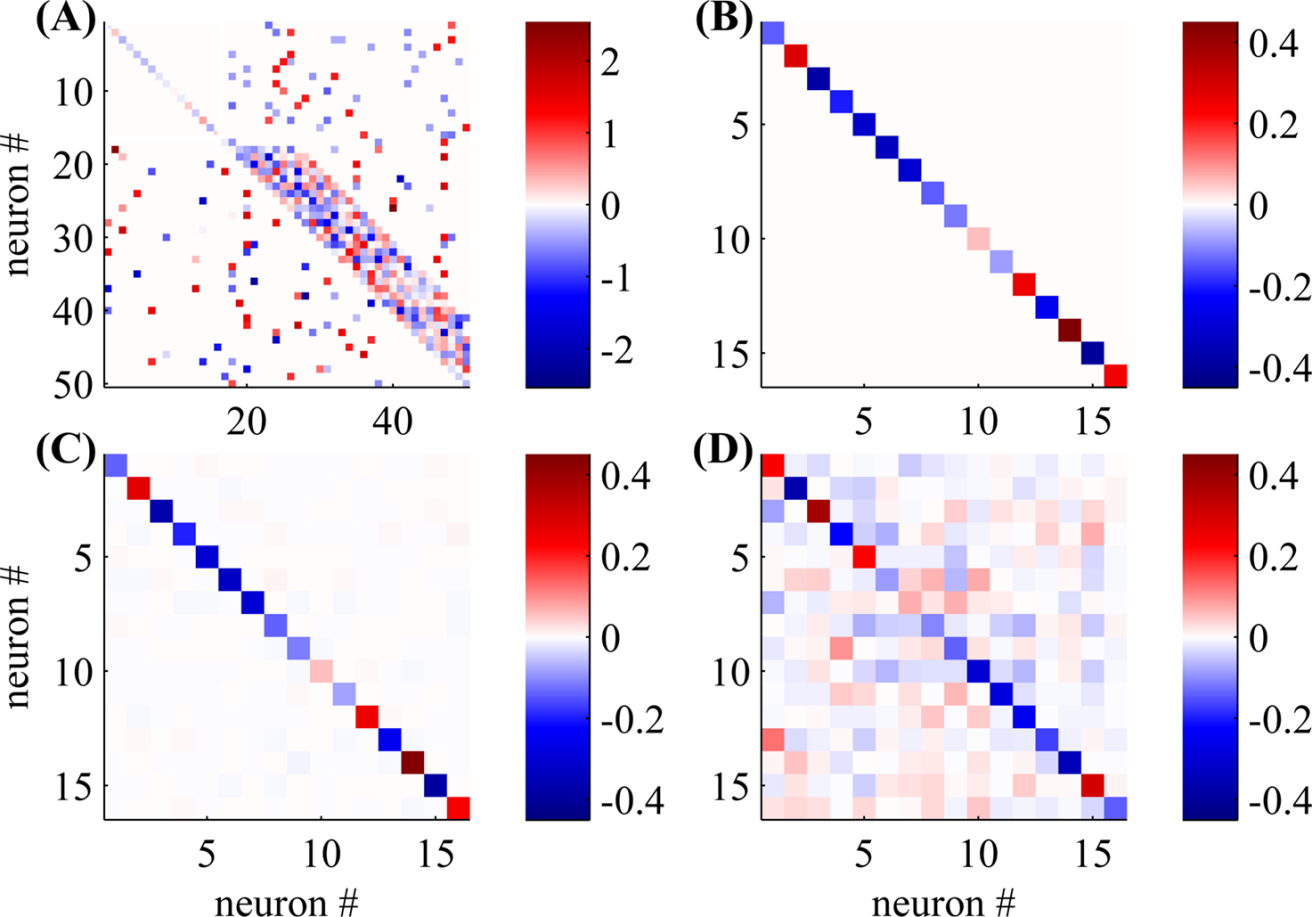
Visualization of the persistence of the common input problem, despite a large amount of spiking data, and its suggested solution—the shotgun approach. **(A)** The true connectivity—the weight matrix **W** of a network with *N* = 50 neurons. **(B)** A zoomed-in view of the top 16 neurons in A (upper left white rectangle in A). **(C)** The same zoomed-in view of the top 16 neurons in the ML estimate of the weight matrix **W** (Eq 25), where we used the shotgun (random blocks) observation scheme on the whole network, with a random observation probability of *p*_obs_ = 16/50. **(D)** The ML estimator of the weight matrix **W** of the top 16 neurons if we observe only these neurons. Note the unobserved neurons cause false positives in connectivity estimation. These “spurious connections” do not vanish even when we have a large amount of spike data. In contrast, the shotgun approach (C), does not have these persistent errors, since it spreads the same number of observations evenly over the network. *T* = 5⋅10^8^, *b*_*i*_ ∼ 𝓝(−0.5, 0.1).

Errors caused by common inputs are particularly troublesome for connectivity estimation, since they can persist even as *T* → ∞. Therefore, for simplicity, we work in a regime where the experiment is long and data is abundant (*T* = 5⋅10^8^ timebins). In this regime, any prior information we have on the connectivity becomes unimportant so we simply use the Maximum Likelihood (ML) estimator. We chose the weight matrix **W** to illustrate a “worst-case” common input condition (Fig 2A). Note that the upper-left third of **W** is diagonal (Fig 2B): *i.e*., neurons *i* = 1, …, 16 share no connections to each other, other than the self-connection terms *W*_*i*, *i*_. However, we have seeded this **W** with many common-input motifs, in which neurons *i* and *j* (with *i*, *j* ≤ 16) both receive common input from neurons *k* with *k* ≥ 17.

If we use a “shotgun” approach and observe the whole network with *p*_obs_ = 16/50 with a fully random observation scheme, we obtain a good ML estimate of the network connectivity, including the 16 × 16 upper-left submatrix (Fig 2C). Now, suppose instead we concentrate all our observations on these 16 neurons, so that *p*_obs_ = 1 within that sub-network, but the other neurons are unobserved. If common input was not a problem, our estimation quality should improve on that submatrix (since we have more measurements per neuron). However, if common noise is problematic, then we will “hallucinate” many nonexistent connections (i.e., off-diagonal terms) in this submatrix. Fig 2D illustrates this phenomenon. In contrast to the shotgun case, the resulting estimates are significantly corrupted by the common input effects.

### 5 Connectivity estimation—quantitative analysis

Next, we quantitatively test the performance of the Maximum A Posteriori (MAP) estimate of the network connectivity matrix **W** using a detailed network model with biologically plausible parameters from the mouse visual cortex. Details on the network parameters, simulation details and definitions of the quality measures are given in section B in S1 Text. We use the inference method described in section 2, with a sparsity inducing prior (section C in S1 Text) on a simulated network with GLM neurons (Eqs (1)–(2)).

**Fig 3:**
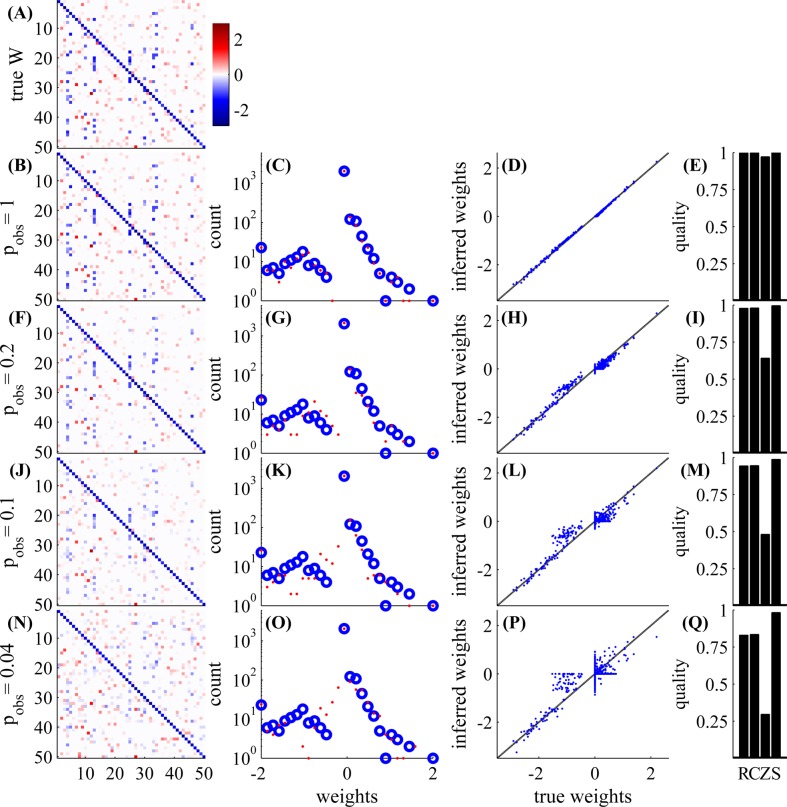
Network connectivity can be well estimated even with low observation ratios. With *N* = 50 neurons, and an experiment length of 5.5 hours, we examine various observation probabilities: *p*_obs_ = 1,0.2,0.1,0.04. *Left* **(A,B,F,J,N)**: weight matrix (either true or estimated). *Middle left* **(C,G,K,O)**: non-zero weights histogram (blue—true, red—estimated). *Middle right* **(D,H,L,P)**: inferred weight vs. true weight. *Right* **(E,I,M,Q)**: quality of estimation—S = sign detection, Z = zero detection, C = correlation, $\text{R}=\sqrt{{\text{R}}^{2}}$ (for exact definitions see Eqs 40–43 in S1 Text); higher values correspond to better estimates. In the first row, we have the true weight matrix **W**. In the other rows we have the inferred **W**—the MAP estimate of the weight matrix with the L1 prior (section C in S1 Text), with *λ* chosen so that the sparsity of the inferred **W** matches that of **W**. Estimation is possible even with very low observation ratios; in the lowest row we observe only 2 neurons out of 50 in each time bin. The weights on the diagonal are estimated better because we observe them more often in double serial scanning scheme (Fig 1I, 1J).

First, in Fig 3, we examine a small GLM network with *N* = 50 observed neurons, with an experiment length of 5.5 hours. As can be seen, the weight matrix can be very accurately estimated for high values of observation probability *p*_obs_, and reasonably well even for low value of *p*_obs_. For example, even if *p*_obs_ = 0.04, and *only two neurons* are observed in each timestep, we get a correlation of *C* ≈ 0.84 between inferred weights and the true weights, and the signs of the non-zero weights are only wrong only for 4 weights (out of 448 non-zero weights). When *p*_obs_ is decreased, the variance of the estimation increases, more weak weights are inferred as zero weights (and vice versa), and we also see more “shrinkage” of the non-diagonal weights (a decreased magnitude of the non-zero weights) due to the *L*1 penalty imposed on them (Eq 47 in S1 Text).

**Fig 4:**
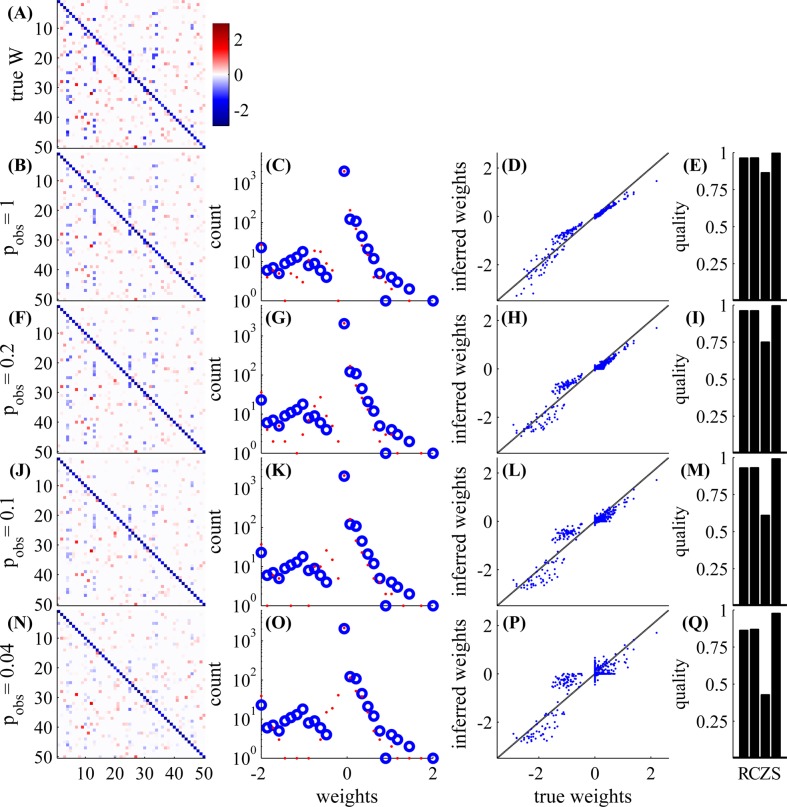
Network connectivity can be reasonably estimated, even with model mismatch. The panels (**A-Q**) are the same as Fig 3, where instead of a logistic GLM (Eq 1), we used a stochastic leaky integrate and fire neuron model (in discrete time). In this model, *V*_*i*, *t*_ = (*γV*_*i*, *t*−1_+(1−*γ*)*U*_*i*, *t*_+*ε*_*i*, *t*_)𝓘[*S*_*i*, *t*−1_ = 0] (**U** defined in Eq 2), *S*_*i*, *t*+1_ = 𝓘[*V*_*i*, *t*_ > 0.5]. We used *ε*_*i*, *t*_ ∼ 𝓝(0,1) as a white noise source. Also, we set *γ* = 20ms^−1^, similar to the inverse of the membrane’s voltage average integration timescale [60]. The weights were estimated up to a global multiplicative constant (resulting from the model mismatch), which was adjusted for in the figure. We conclude that our estimation method is robust to modeling errors, except perhaps the diagonal weights—their magnitudes were somewhat over-estimated due to the reset mechanism (which effectively increases self inhibition).

In Fig 4 we demonstrate that our method works well even if the neuron model is not a GLM, as we assume, but a Leaky Integrate and Fire (LIF) neuron model (Fig 4). The model mismatch results in a weight mismatch by a global multiplicative constant, and in a worse estimate of the diagonal weights, due to the hard reset in the LIF model. Besides these issues, inference results are both qualitatively and quantitatively similar to results in the GLM network

**Fig 5:**
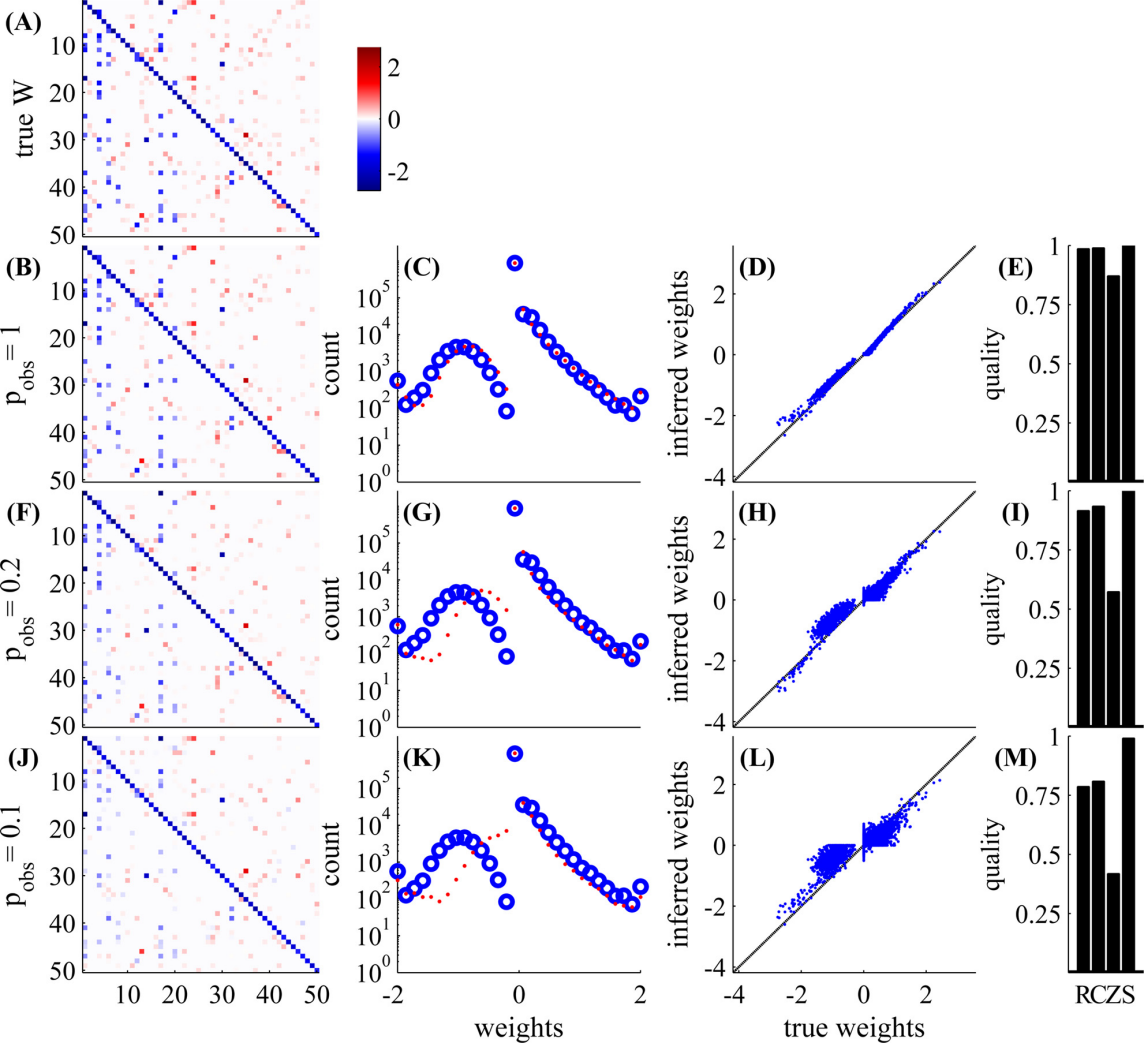
Network connectivity can be well estimated even with a large network. The panels (**A-M**) are arranged in columns as in Fig 3, except now we have *N* = 1000 observed neurons and additional 200 unobserved (this is the same simulation as in Fig S1), and *p*_obs_ = 1, 0.2 and 0.1. In the left (A,B,F,J) and middle right columns (D,H,L) we show a random subset of 50 neurons out of 1000, to improve visibility. The other columns show statistics for all observed neurons. On a standard laptop, the network simulation takes about half an hour, and the connectivity estimate can be produced in minutes. Therefore, our algorithm is scalable, and much faster than standard GLM based approaches, as we explain in section 6.2.

**Fig 6:**
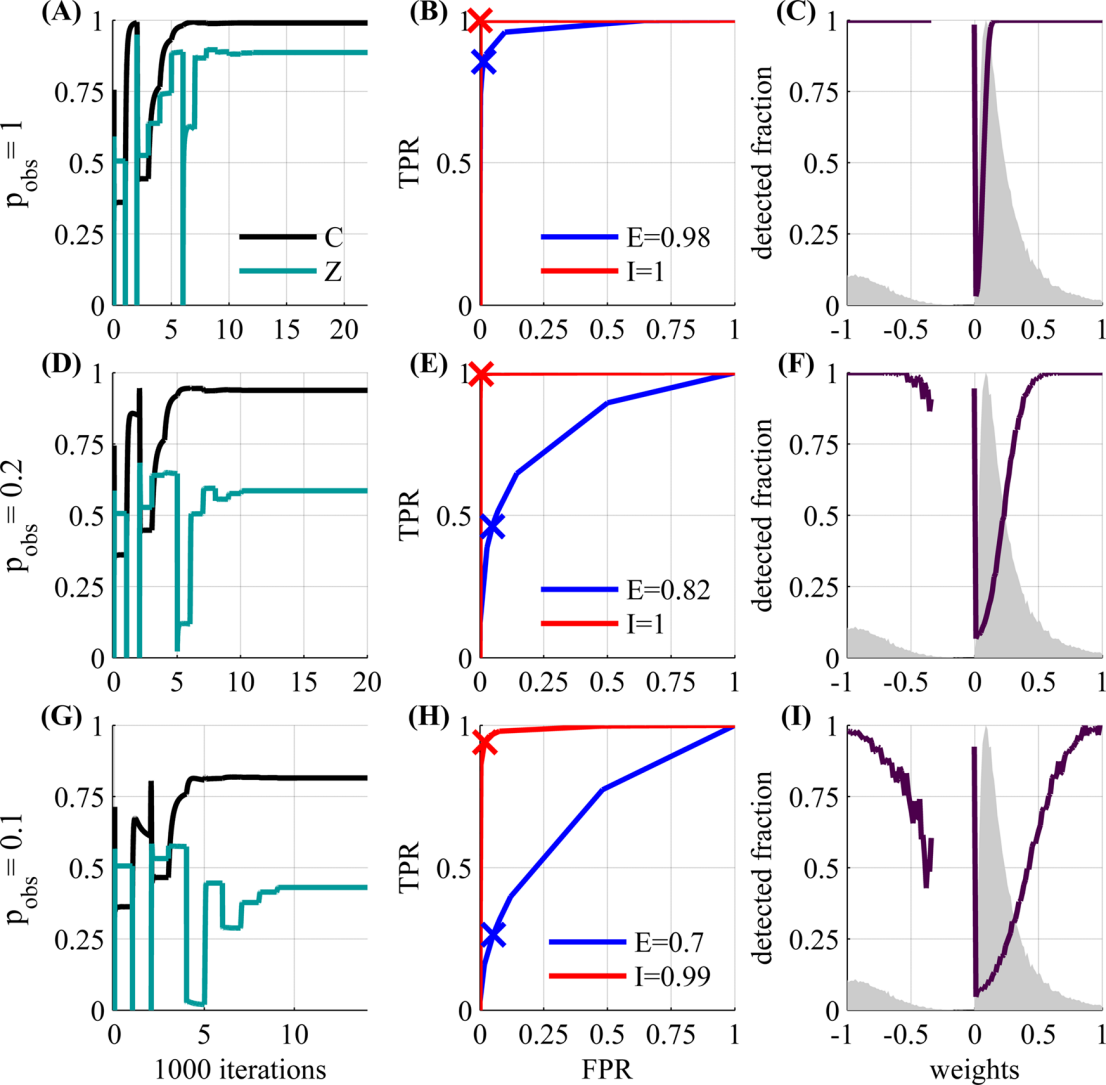
Statistical Analysis. We use the same network as in Fig 5 with *N* = 1000 and *p*_obs_ = 1, 0.2 and 0.1. *Left* **(A,D,G)**: convergence of performance. Recall that we use the FISTA algorithm (section C.1 in S1 Text) inside an outer loop that sets the regularization parameter according to sparsity (section C.2 in S1 Text). Therefore convergence is non-monotonic, and “jumps” each time the parameter is changed. Each time this happens, it takes about a thousand iterations until convergence. *Middle* **(B,E,H)**: Receiver Operating Characteristic (ROC) curve, showing the trade-off between the false positive rate and the true positive rate (FPR and TPR, Eqs 44 and 45 in S1 Text, receptively) in detecting excitatory (blue) or inhibitory weights (red)—*i.e*., inferring a non zero weight, with the right sign. The curve illustrates the classification performance as the discrimination threshold is varied by changing *λ*, the L1 regularization parameter (Eq 46 in S1 Text). The ‘×’ marks the performance for *λ* chosen by our algorithm. For each case (excitatory/inhibitory), the measures *E* and *I* are the area under the curve—values close to 1 (0.5) indicate good (bad) performance. Performance is significantly better for the inhibitory weights, since they are typically stronger, and we can more easily distinguish non-zero weights. We see this explicitly on the *Right* **(C,F,I)**: Magenta line—fraction of weights detected with the correct sign (-1,0, or 1) as a function of weight value. Line stops if less than 30 weight values exist in that range. For clarity, we added the non-zero weight distribution (shaded gray area, scaled to fit panel) and zoomed on the range [−1, 1]. Weights with magnitude larger then 1 were perfectly detected. Small weights are harder to detect at low *p*_obs_.

In Fig 5, we examine another GLM network with *N* = 1000 observed neurons, which is closer to the scale of the number of recorded neurons in current calcium imaging experiments (see activity simulation in Fig S1 S1 Text). The experiment duration is again 5.5 hours. Results are qualitatively the same as the case of *N* = 50, except performance is somewhat decreased (as we have more parameters to estimate). Additional information is available in Fig 6. On the left (A,D,G), we see that the algorithm converges properly to a single solution. In the middle panels (B,E,H), we see that for *p*_obs_ = 1 we have very good performance (in terms of area under the ROC), but this performance declines for the excitatory weights as *p*_obs_ decreases. The inhibitory weights are correctly detected much better than the excitatory weights. This is because most excitatory weights are much weaker, as can be seen on the right column (C,F,I). In that column, we observe that strong weights are more easily detected than weak weights. Specifically, around the median of the excitatory weight distribution (0.178), we detected 99.9%, 34.9% and 16.1% of all the weights, when *p*_obs_ = 1,0.2 and 0.1, respectively.

**Fig 7:**
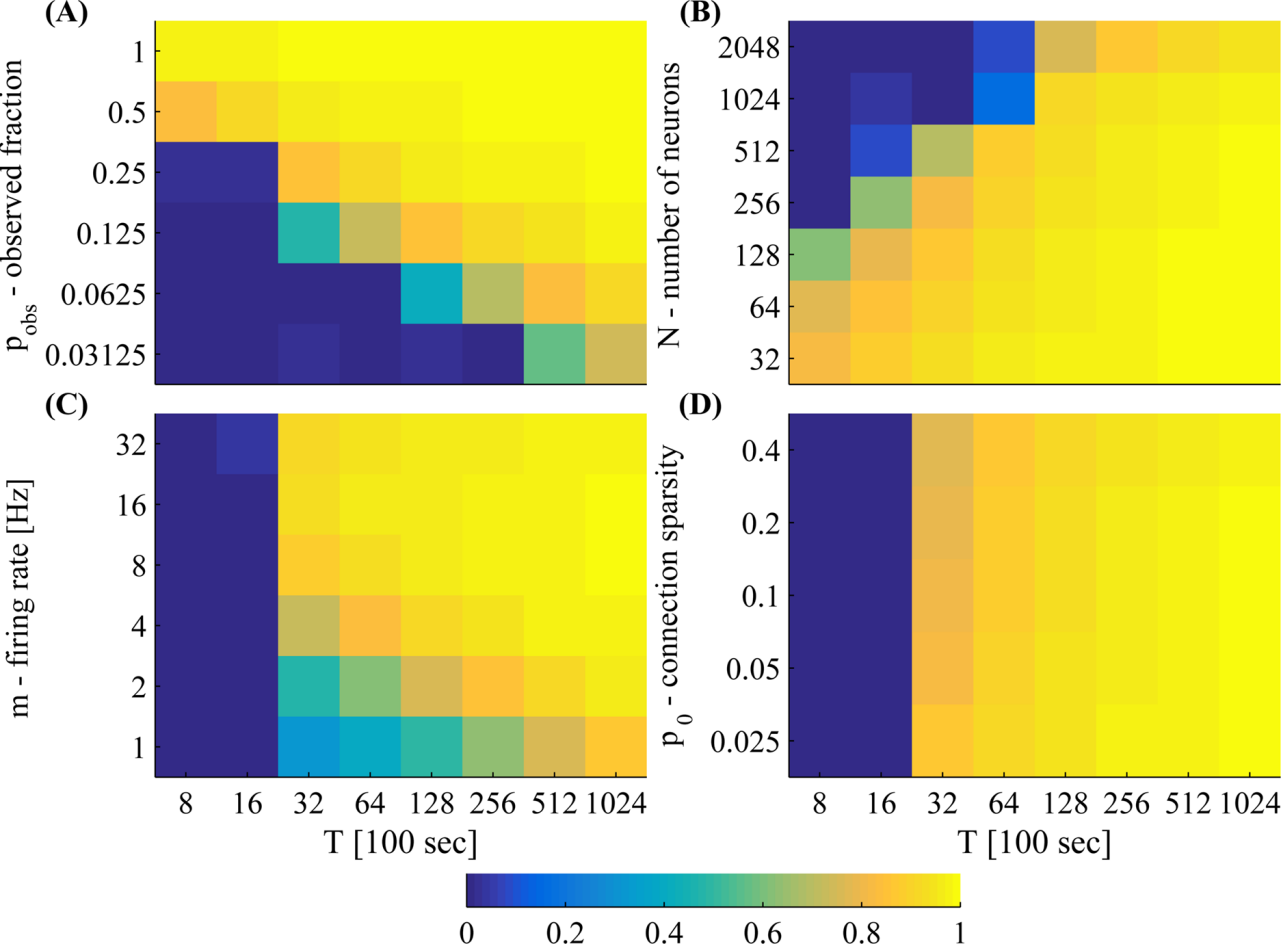
Parameter scans show that *C*, the correlation between true and estimated connectivity, monotonically increases with *T* in various parameter regimes. We scan over **(A)** observation probability *p*_obs_, **(B)** network size *N*, **(C)** mean firing rate *m* (similar to the firing rate of the excitatory neurons—inhibitory neurons fire approximately twice as fast), and **(D)** connection sparsity parameter *p*_0_ (which is proportional to actual connection sparsity *p*_conn_—see Eq 37 in S1 Text) and experiment duration *T*. Other parameters (when these are not scanned): *p*_obs_ = 0.2, *N* = 500.

Next, in Fig 7 we quantify how inference performance changes with parameters. We vary the number of neurons, *N*, observation probability *p*_obs_, mean firing rate *m* and connection sparsity *p*_conn_. For the given parameters *N*, *p*_obs_ and *m*, performance monotonically improves when *T* increases. These scans suggest we can maintain a good quality of connectivity estimation for arbitrarily large or small values of *N* or *p*_obs_, respectively—as long as we sufficiently increase *T*. Note there is a lower bound on *T*, below which estimation does not work. Looking at Fig 7, we find that approximately, this lower bound scales as

$$\begin{array}{c} T\propto \frac{N}{p_{\text{obs}}^{2}}\;. \end{array}$$ (18)

Above this lower bound, estimation quality gradually improves with *T*. Moreover, in order to maintain good estimation quality (up to some saturation level) above this bound, *T* should be scaled as

$$\begin{array}{c} T\propto \frac{N}{p_{\text{obs}}^{2}m^{2}}\;. \end{array}$$ (19)

This scaling can be explained intuitively. Our main sufficient statistic is the partially observed spike covariance ${\tilde{\text{Σ}\;}}^{\left(k\right)}$ (Eq 17). Each component (*i*, *j*) of ${\tilde{\text{Σ}\;}}^{\left(k\right)}$ contains a sum of all the observed spike pairs (*T*⟨*O*_*i*, *t*_ *O*_*j*, *t*−*k*_ *S*_*i*, *t*_ *S*_*j*, *t*−*k*_⟩_*T*_) divided by the number of observed neurons (*T*⟨*O*_*i*, *t*_ *O*_*j*, *t*−*k*_⟩_*T*_). The total number of observed neuron pairs is approximately $NTp_{\text{obs}}^{2}$ (ignoring observation correlations), and the total number of observed spike pairs is approximately $NTp_{\text{obs}}^{2}m^{2}$ (ignoring spike correlations, and assuming the firing rate is not very high), where *T* is measured in time bins. The total number of components in ${\tilde{\text{Σ}\;}}^{\left(k\right)}$ is *N* ^2^. Therefore, in each component of ${\tilde{\text{Σ}\;}}^{\left(k\right)}$, the average number of observed neuron pairs is $Tp_{\text{obs}}^{2}/N$, while the average number of observed spike pairs is approximately $Tp_{\text{obs}}^{2}m^{2}/N$ (except on the diagonal of **Σ** ^(0)^, where we have *Tp*_obs_/*N* neuron pairs and *Tp*_obs_ *m*/*N* spikes). We conclude that the number of both observed neuron pairs and spike pairs must be above a certain threshold so that inference will be able to work properly. Above these thresholds, performance improves further when *p*_conn_ is decreased (Fig 7), as this reduces the effective number of parameters we are required to estimate. For analytic results on this issue see [28].

**Fig 8:**
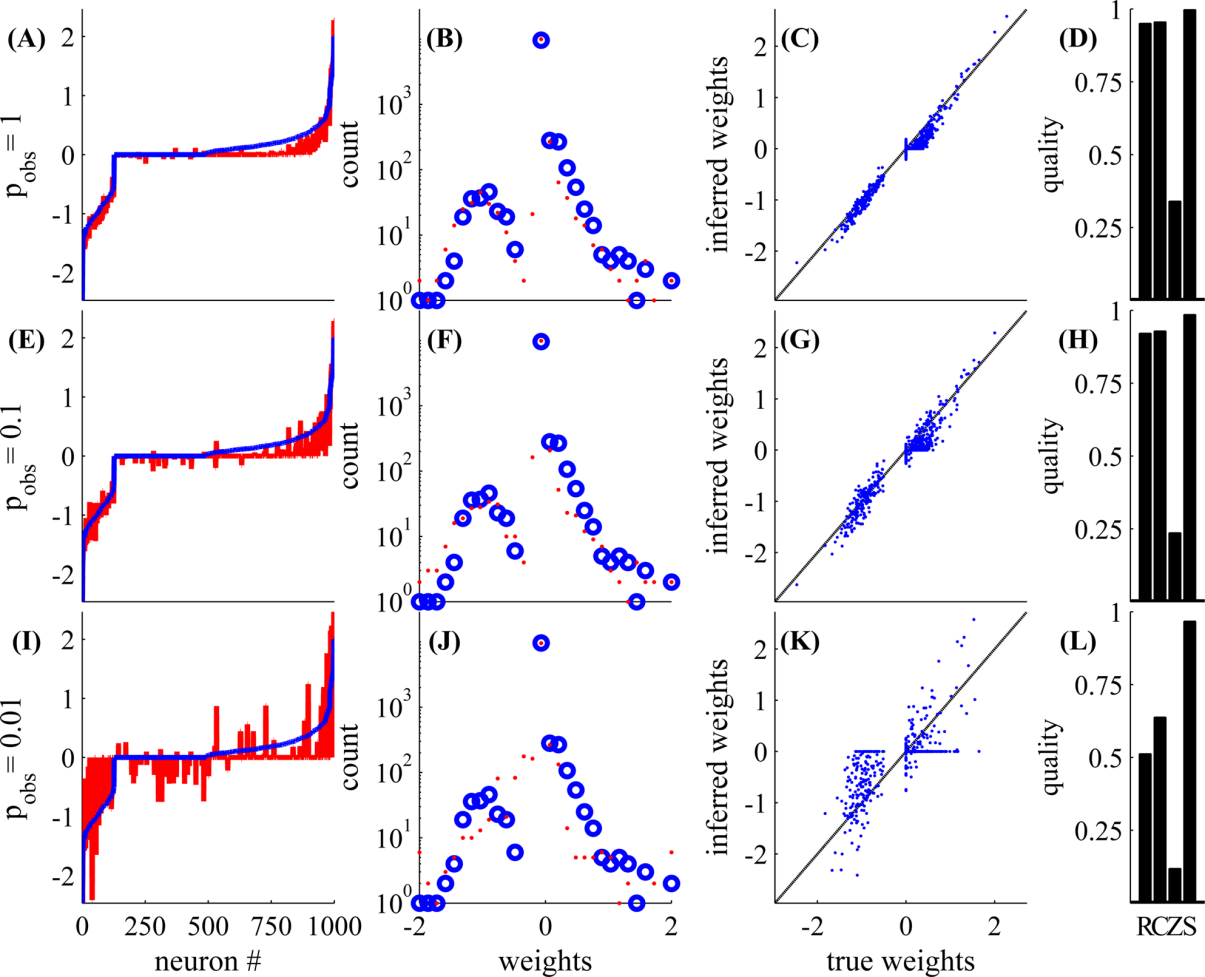
Inferring input connectivity to a single neuron with many inputs and low observation ratios. The panels **B-D**,**F-H**, and **J-L** are arranged in columns as in Fig 3. In the left column **(A,E,I)** we show a sample of 1000 input weight values from the true (blue) and inferred weights (red), sorted according to the value of the true weights. In the other panels, we show all the weights. We have *N* = 10626 observed inputs, 968 of which have non-zero weights. The output neuron is always observed, while 83% of the input neurons are only partially observed with *p*_obs_ = 1,0.1,0.01. The rest are never observed. Other network parameters are the same as before (*e.g*., *T* = 5.5 hours), and the firing rate of the output neuron was 2.8Hz. More implementation details in S1 Text, section B.2.

If our goal is to infer all the input connections of a single neuron, then performance can be significantly improved if we always observe the output of that neuron. This is demonstrated in Fig 8. In this figure, we examine a single neuron with *O*(10^4^) observed inputs, *O*(10^3^) of which are non-zero (implementation details in S1 Text, section B.2). The inputs are partially observed (with *p*_obs_ = 1,0.1,0.01), but we always observe the output neuron. Therefore, the average number of observed neuron pairs and spike pairs in **Σ** ^(1)^ is increased to *NTp*_obs_ and *NmTp*_obs_, respectively. This can improve the scaling relations in Eqs (18) and (19) to *T*/*p*_obs_ and *T*/(*p*_obs_ *m* ^2^), respectively, if the off-diagonal terms of **Σ** ^(0)^ are not too strong (since the number of observations for these components still scales with $\propto p_{\text{obs}}^{2}$). Thus, in Fig 8, we see that even when *p*_obs_ = 0.01 it is still possible to estimate strong weights with some accuracy, despite the large number of connections.

### 6 Expected LogLikelihood-based estimation: accuracy and speed

#### 6.1 Statistical efficiency

**Fig 9:**
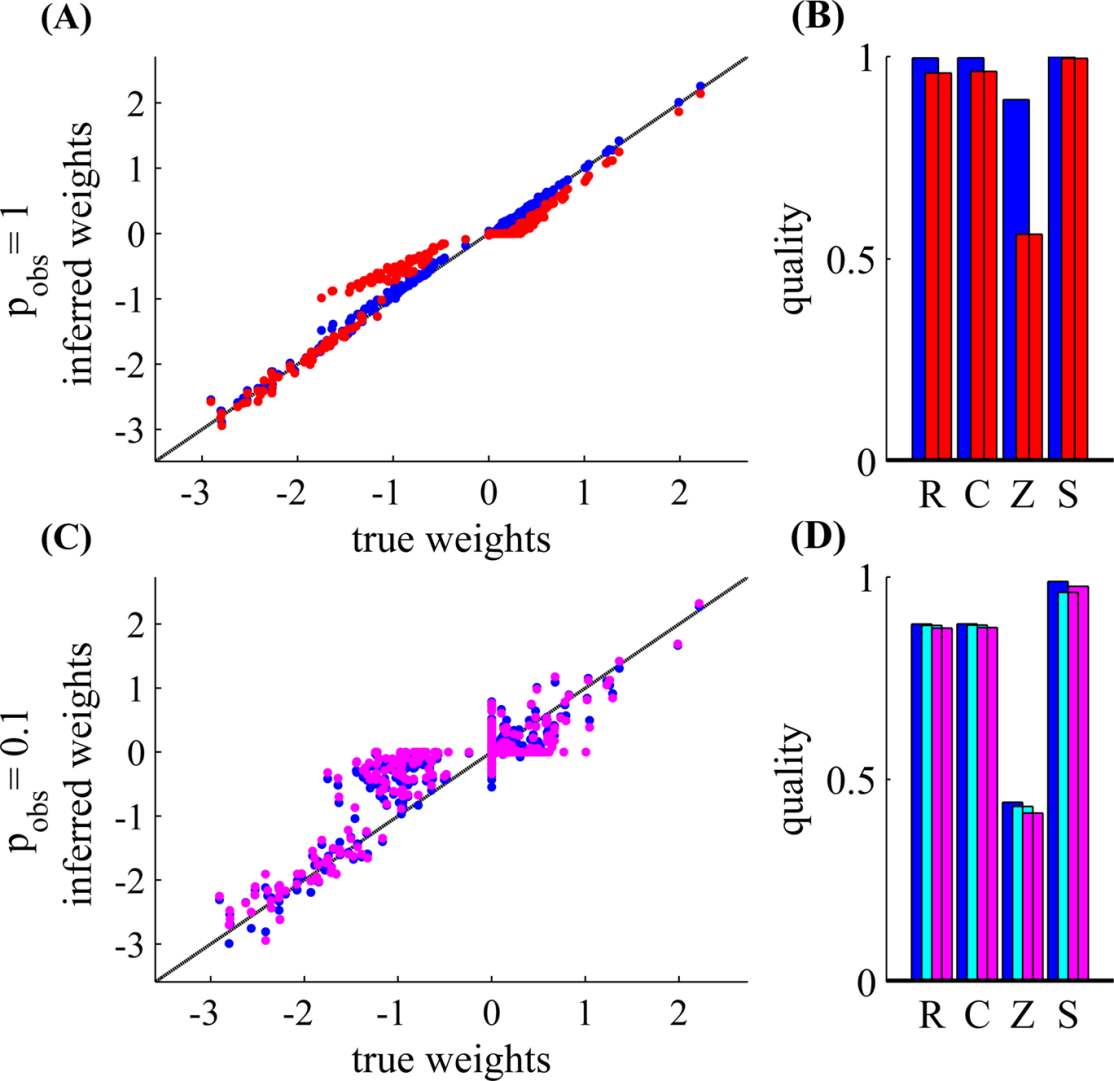
Expected LogLikelihood (ELL) based estimation is statistically efficient. *Top* **(A,B)**: The ELL-based method (blue) compares favorably to the standard MAP estimate (red) when spikes are fully observed (using the same L1 prior). *Bottom* **(C,D)**: We compare the ELL based method (blue) to the Expectation Maximization (EM) approach, when only 10% of the spikes are observed. We show the results after one (cyan) and two (magenta) EM steps. The EM steps do not improve over the ELL-based method. Parameters: *N* = 50, *T* = 1.4 hours. For the Gibbs sampling we used a single sample after a burn-in period of 30 samples, as we used in our EM simulations without the ELL-based initialization (section E S1 Text).

The results of the previous section (mostly, Fig 3, for *p*_obs_ = 1, as well as our parameter scans) demonstrate numerically that our Expected LogLikelihood-based (ELL) estimation method is effective given sufficiently large observation times *T*. However, it is still not clear if our approximations hurt the statistical efficiency of our estimation. Specifically, can we get a significantly smaller error with the same *T*, if we did not use any approximations? We give numerical evidence in Fig 9 that this is not the case. All the parameters are as described in S1 Text, section B.1, except we used a random blocks observation scheme (see Fig 1G, 1H) and did not have any unobserved neurons.

First, we examine the case in which all the spikes are observed (*p*_obs_ = 1). We compare our ELL-based estimate with standard MAP optimization of the full likelihood (with the same L1 prior). We implement the latter by plugging the weight gradients of the loglikelihood (Eq 7) in the same optimization algorithm we use for the ELL-based estimate (section C.1 in S1 Text), together with the bias gradients. As can be seen in Fig 9A, 9B, the approximate ELL-based MAP estimate (blue) actually slightly outperforms the accurate MAP estimate (red, which exhibits more shrinkage). These results support the validity of our approximations (See [13] for further discussion of how the ELL approximation can in some cases improve the MAP estimate).

Next, we demonstrate numerically that we can safely ignore missing spikes without increasing estimation error, when *p*_obs_ < 1. Specifically, we want to verify the efficiency of using the “partial” empirical moments ($\tilde{\text{m}}$ and $\tilde{\text{Σ}\;}$) instead of the full sufficient statistics (**m** and **Σ**), as detailed in section 3.6. These full sufficient statistics can be potentially inferred “correctly” using Bayesian inference techniques such as Markov chain Monte Carlo (MCMC) (section E in S1 Text). In Fig 9C, 9D, we demonstrate that inferring these missing spikes does not improve our estimation. We compare the ELL-based estimate (blue), to the estimate produced by initializing with the ELL-based estimate and then performing one (red) and two (magenta) Expectation-Maximization (EM) steps [29]. In more detail, to perform the first EM step we first estimate **W** using the ELL-based method, then Gibbs sample the missing spikes (section E.1.1 in S1 Text), assuming this **W** is the true connectivity, and then infer **W** again using the ELL-based method. For the second step, we initialize **W** with the first step, Gibbs sample the missing spikes, and infer **W**, again using the ELL-based method. This Monte Carlo EM procedure should converge to a local optimizer of the full log-posterior, assuming a sufficient number of MCMC samples are obtained in each iteration [29]. Nonetheless, empirically, we see that these EM steps do not help to improve estimation quality here. In addition, using the standard MAP estimate of **W** (instead of the ELL-based estimate) in the EM steps does not qualitatively change these results (data not shown).

#### 6.2 Computational efficiency

An important advantage of the ELL-based method is that it enables extremely fast MAP estimation of the weights (Eq 5). In the standard MAP estimate, we need to calculate all the *N* ^2^ components of the gradient of the original loglikelihood (Eq 7). In total, this requires *O*(*N* ^3^ *T*) operations in each iteration of the optimization procedure. We also find *O*(*N* ^3^ *T*) operations-per-step in other standard estimation methods we tested for ***W*** (MCMC and variational Bayes, see section E.1.2 in S1 Text). In contrast, in the ELL-based method, the first step is to calculate $\tilde{\text{m}}$ and $\tilde{\text{Σ}\;}$, which takes *O*(*N* ^2^ *T*) operations, but we only need to do this once (this usually takes much less time than the simulation of the network activity, which also takes *O*(*N* ^2^ *T*) operations). Once these are calculated, we need only *O*(*N* ^3^) operations to calculate the loglikelihood (or its gradient) in each iteration of the optimization algorithm. This results in orders of magnitude improvements in estimation speed over the standard MAP estimate from the original loglikelihood.

For example, in the simulation we show in Fig 9A, 9B, (where *N* = 50 and *T* = 5⋅10^5^ time bins) it takes about 11 seconds to run the optimization algorithm using the ELL-based method: approximately one second to calculate the empirical moments, and 10 seconds for the optimization algorithm to converge. A single step of calculating the gradient and updating the weights (in the internal FISTA loop) took 0.002 sec. A similar step took 0.7 sec in the standard MAP estimation. In total, the algorithm took about 4 hours to converge (taking more iterations than the ELL-based method).

As another example, in [30], it takes *O*(10^5^) CPU hours using a computer cluster to estimate the connectivity of a network (where *N* = 1000 and *T* = 3.6 ⋅ 10^7^ time bins). In our case (where *N* = 1000 and *T* = 2 ⋅ 10^6^ time bins), a similar simulation on a standard laptop (Fig 5) takes about half an hour to generate the spikes, together with the sufficient statistics, and a few more minutes to perform the estimation for a given prior distribution. While our model is slightly simpler than that of [30], most of this massive improvement in speed is due to the differences in the inference methods used.

Lastly, we note that Gibbs sampling the spikes also requires *O*(*N* ^3^ *T*) operations in each time step (Eq 78 in S1 Text). For example, in the simulation behind Fig 9C, 9D, each Gibb steps for all the spikes took about *2.5* minutes. All the steps took in total about 80 minutes. Therefore, ignoring the missing spikes, instead of sampling them, greatly improves computational speed.

### 7 Fluorescence-based inference

**Fig 10:**
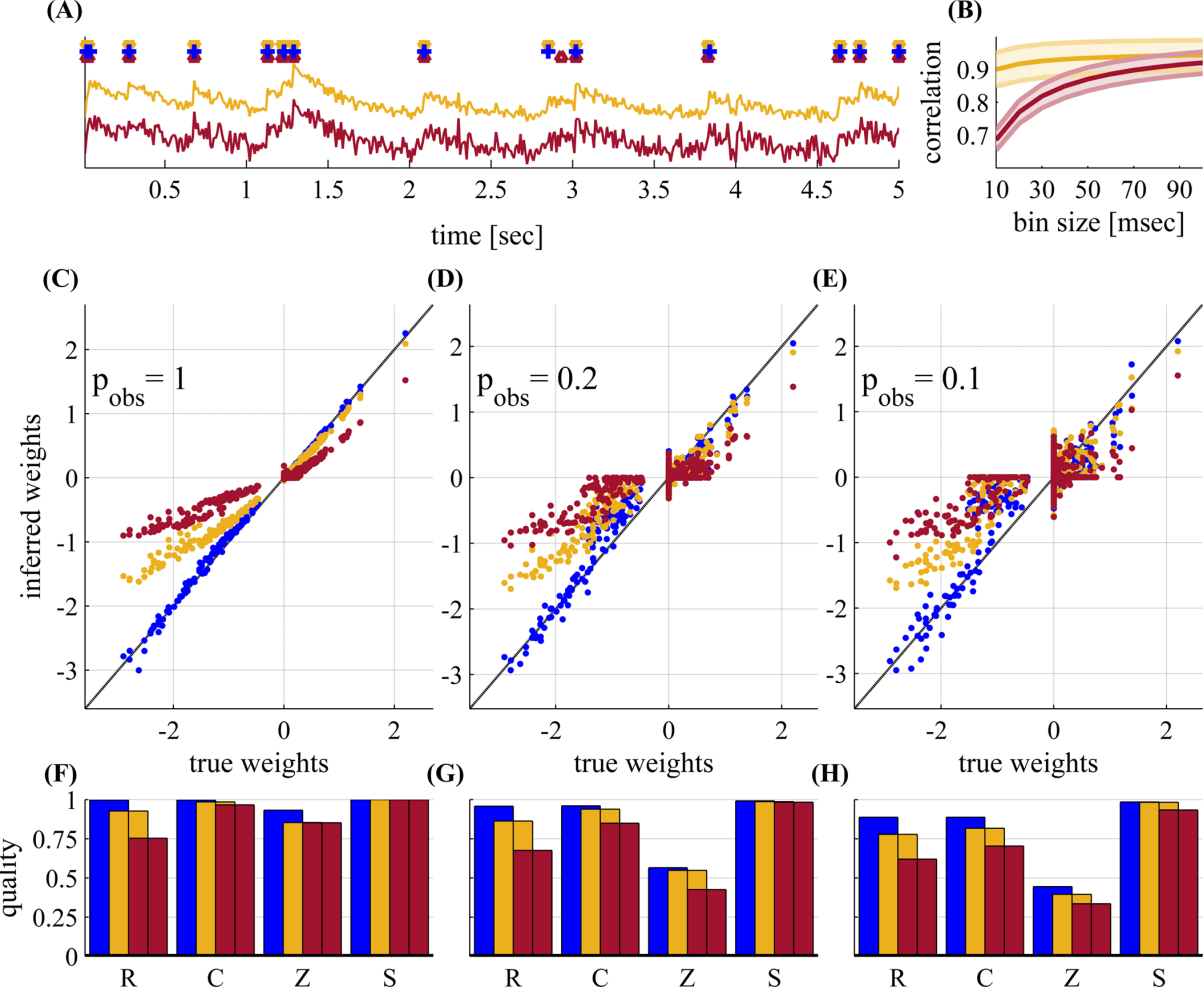
Inferring connectivity from fluorescence measurements from a network with *N* = 50 observed neurons—for different noise regimes: none (blue), low (yellow, snr = 0.2) and high (brown, snr = 0.4). **(A)** A short sample showing the fluorescence traces, for both noise regimes. On top we show the actual spikes (blue cross), and inferred spikes for low / high noise (yellow/brown triangles). **(B)** Correlation (population mean ± std) between actual and inferred spikes,for both low and high noise regimes. We bin the spikes (both actual and inferred) at various time bin sizes (x axis) and calculate the correlation using the definition of *C* (Eq 41, only for spikes instead of weights). Spikes are reasonably well estimated, given the noisy fluorescence traces. **(C-E)** Estimated weights vs. true weights for *p*_obs_ = 1, 0.2 and 0.1. **(F-H)** Quality of inference for the C-E, respectively. Blue—spikes are directly measured, Yellow / brown—spikes are inferred from the respective fluorescence traces. Inhibitory weights exhibit more “shrinkage” due to their higher firing rate, which makes it harder to infer spikes from fluorescence. The mean firing rate is 3Hz, and *T* = 5.5 hours.

In a real imaging experiment, we would not have direct access to spikes, as we have assumed for simplicity so far. Next, we test the estimation quality when we only have direct access to the fluorescence traces of activity (Fig 10A). The fluorescence traces were generated using a model of GCaMP6f calcium fluorescence indicator. Implementation details are described in section B.3 in S1 Text. As can be seen in Fig 10A, 10B, our spike inference algorithm works reasonably well, both in high and low noise regimes. We then infer network connectivity both from the inferred spikes and the true spikes. As can be seen in Fig 10C-10H, using the inferred spikes usually somewhat reduces estimation performance. This is due to the temporal inaccuracy in the spike estimation. For example, in the inhibitory neurons, the higher firing rates result in more missing spikes in the inference. This causes shrinkage in the magnitude of the inferred weights, since the cross-correlation is weakened by these missing spikes. Combining this information into the inference algorithm (as in [9]), it may be possible to correct for this; we have not pursued this question further here. However, even at low observation probabilities (*p*_obs_ = 0.1), strong weights are inferred reasonably well, and the sign of synapse is usually inferred correctly for almost all nonzero weights. Therefore, weight inference is still possible at low firing rates, using current generation fluorescence imaging methods.

## Discussion

### 8 Previous works

Neural connectivity inference has attracted much attention in recent years. One approach to this problem is direct anatomical tracing [31, 32]. However, this method is computationally challenging [33]; moreover, the magnitudes of the synaptic connections (which also vary over time [34]) currently cannot be inferred this way. Another approach, on which we focus here, aims to infer the synaptic connectivity from neural activity. This activity can be either action potentials (“spikes”) [16, 35–37] or calcium fluorescence traces [9, 18, 38–40] which are approximately a noisy and filtered version of the spikes.

Various inference procedures have been suggested for this purpose. Some works use model-free empirical scores [38, 40, 41]. Others assume an explicit generative model for the network activity [16, 30, 35–37], and then infer connectivity by estimating model parameters. So far, only few works have validated the connectivity estimate with some form of “ground truth”. Gerhard et al. [19] inferred small scale anatomical connectivity, comparing different methods. A Generalized Linear Model (GLM) approach was successful, while linear models and model-free approaches failed. Volgushev et al. [42] estimated the weights of fictitious synapses (injected current). Again, a GLM-based approach outperformed simple correlation-based approaches. Lastly, Latimer et al. [43] was able to infer the magnitude of intracellular synaptic conductances, using a modified GLM. These results indicate that a GLM-based approach should be the method of choice for estimating synaptic connectivity.

The task of inferring synaptic connectivity is severely hindered by technical limitations on the number of neurons that can be simultaneously observed with sufficient quality. Typically, the scanning speed of the imaging device is limited, so we cannot cover the entire network with a high enough frame rate and signal-to-noise ratio to infer spikes from the observed fluorescence traces. Previous studies indicate that at low frame rates (below 30Hz [9]), synaptic connectivity cannot be inferred. In such low frame rate regimes, one may use spike correlations or simple dynamical systems as a coarse measure of effective connectivity (*e.g*., [44]), but such measures are not claimed to predict synaptic connectivity, only provide a statistical description of the network dynamics.

Therefore, common approaches to infer connectivity of a neural network focus all the observations in one experiment on a small part of the network, in which all neurons are fully observed at a high frame rate. However, unobserved input into this sub-network can generate significant error in the estimation, and this error does not vanish with longer experiments. Various works aimed to deal with this persistent error: [17] inferred connectivity in a simulated two-neuron network in which one neuron was never observed; [45] inferred connectivity in a simulated network with two observed neurons and an unobserved common input; [46] inferred unobserved common input in an experimentally recorded network of 250 neurons using a GLM network with latent variables; [47] inferred connectivity in a simulated network with 100 neurons where 20−50 were never observed, with a varying degree of success.

### 9 The shotgun approach

To help deal with the “common input” problem, we propose a “shotgun” approach, in which we reconstruct network connectivity by serially observing small parts of the network—where each part is observed at a high frame rate for a limited duration. Thus, despite the limited scanning speed of the imaging device, by using this method, we can extend the number of the neurons covered by the scanning device and effectively decrease the number (and therefore the effect) of unobserved common inputs. Additionally, as only a small part of the network is illuminated together, this method can potentially reduce phototoxicity and photobleaching, and allow long, possibly chronic [48], imaging experiments.

#### 9.1 Inferring correlations

Though our goal is to infer synaptic connections, we first discuss the closely related goal of inferring correlations between neurons. It is straightforward to infer these correlations from sub-sampled shotgun data when all neuron pairs can be observed together for long enough durations. We simply have to “ignore” any unobserved activity (section 3.6). We therefore suggest several observation schemes that might be used to eventually observe a much larger fraction of neuron pairs in the network (section 3). For example, we show that this can be done using two serial scanners with incommensurate periods (Fig 1I, 1J). If two scanning systems are combined on the same microscope, it can increase the effective frame rate above the critical 30Hz level [9] and allow successful weight reconstruction given long enough experiments. Alternatively, if we can use two moving microscopes to implement this scheme [27], the “effective field of view”, could be expanded to any region that is not visually obstructed (such as deep regions in the tissue). This expansion can be arbitrarily large, again, as long as the experimental duration is long enough to compensate.

It may be also possible to infer correlations even if not all neuron pairs are observed (e.g., in a serial scanning scheme). For example, the methods discussed in [44, 49] might be helpful, if the fraction of observed neurons is not too low. In [44], in which experimentally recorded spikes are divided into two minimally overlapping blocks, the covariance matrix could only be accurately completed if more than 60% of the neurons were observed in each block (so, in total, 68% of all neuron pairs). Another covariance matrix completion method loosely requires that the size of the overlapping regions between the blocks must be larger than the rank of the full matrix [49]. It is not yet clear when these conditions apply in a physiologically relevant regime. And so, it remains to be seen if such methods could be used when only small fraction of all neurons is observed in each block.

#### 9.2 Inferring connections

As we discussed in the previous section, it relatively straightforward to infer neuronal correlations, given enough observed neural pairs. It is also relatively easy to infer a linear-Gaussian model of the network activity with missing observations [44, 50], since we can analytically integrate out any unknown observations. However, as mentioned earlier (section 8) when inferring actual synaptic connectivity from real data, correlation and linear-based methods are inferior to a GLM-based approach.

Connectivity estimation with missing observations in a GLM is particularly challenging. Standard inference methods (maximum likelihood or maximum a posteriori) cannot be used, since the GLM likelihood cannot be evaluated without first inferring the missing spikes. However, exact Bayesian inference of the unobserved spikes is generally intractable. Therefore, previous works approximated the unobserved spikes through sampling [9, 17, 51], using Markov Chain Monte-Carlo (MCMC) methods on a GLM. However, such methods typically do not scale well for large networks. In fact, even if all the spikes are observed, inferring network connectivity using GLMs is very slow—taking about 10^5^ CPU hours for a network with a thousand neurons in the recent work of [30].

In order to infer connectivity from this type of sub-sampled data we developed an Expected LogLiklihood (ELL) based method, which approximates the loglikelihood and its gradients so they depend on the spikes only through easily estimated second order statistics. By ignoring missing spikes in these statistics, we can infer neural network connectivity even when the spike data is (heavily) sub-sampled. This way we avoid the task of inferring the unobserved spikes, which requires computationally expensive latent variable approaches (section E) in S1 Text, as in [9, 17, 39, 51]. Even when all neurons are observed, the computational complexity drastically improves (section 6.2)—from *O*(*N* ^3^ *TK*) in standard algorithms, to *O*(*N* ^3^ *K*+*N* ^2^ *T*) in the ELL-based method, where *K* is the number of iterations in the algorithm (*K* did not increase in the ELL-based estimation).

#### 9.3 Numerical results

We demonstrate numerically (section 3.6) that such a double serial scanning method can be used to estimate the synaptic connectivity of a spiking neural network with connectivity roughly similar to that of the mouse visual cortex. We show that the inference is possible even if the spike data is sub-sampled at arbitrarily low observation ratios (*e.g*., 10% in a network model with *N* = 1000 neurons, Figs 5 and 6, or 1% in single neuron model with *O*(10^4^) inputs, Fig 8); if the actual neuron model is not a GLM (a LIF model, Fig 4); and if fluorescence traces are observed instead of spikes (Fig 10). We perform parameter scans to examine the robustness of our method, and find the amount of data required for accurate shotgun reconstruction (Fig 7). Additionally, we confirm the accuracy and efficiency of our ELL-based method, in comparison to existing methods (Fig 9).

These results indicate that by using the shotgun observation scheme, we can remove the persistent bias resulting from the common input problem (Fig 2). Therefore, the limited scanning speed of imaging devices is not a fundamental obstacle hindering connectivity estimation. A complete removal of the bias is possible only if all the neurons in the network are observed together with all inputs to the network for a sufficient length of experimental time. However, in most experimental setups, some neurons will never be observed. Therefore, some persistent bias may remain. We modelled such a small bias in all simulations by adding a small number of neurons which are never observed. As we demonstrate numerically, this did not have a strong effect on our results. Stronger common inputs may require the incorporation of latent variables in the model, as in [46]; this is conceptually straightforward, and is an important direction for future research.

Clearly, the most important test for a connectivity inference method is on experimental data. Typically, on real data, performance is almost never as good as in simulations. Moreover, our numerical results suggest that, though our method is clearly much faster and more scalable than previous approaches, it still requires a substantial amount of data (hours). For low amounts of data (e.g., due to low observation ratios) it is likely to capture only the strongest connections accurately (Fig 6). These limitations are not properties of the method, but rather properties of the problem at hand and the type and amount of data typically available. For example, it was previously demonstrated in [42] that a significant amount of data is required to infer weak weights. However, there are a few potential extensions to the inference method that may significantly improve performance, as we explain next.

### 10 Challenges and future directions

We showed here that the proposed method is capable of incorporating prior information about the sparsity of synaptic connections. More specific information could be included. An abundance of such prior information is available for both connection probabilities and synaptic weight distributions as a function of cell location and identity [52]. Cutting edge labeling and tissue preparation methods such as Brainbow [53] and CLARITY [54] are beginning to provide rich anatomical data about “potential connectivity” (*e.g*., the degree of coarse spatial overlap between a given set of dendrites and axons) that can be incorporated into these priors. Exploiting such prior information can significantly improve inference quality, as demonstrated in previous network inference papers [9, 17, 55]. For example, by adjusting the L1 regularization parameters, we can reflect such additional priors: that the probability of having a connection between two neurons typically decreases with the distance between two neurons, and that it is affected by the neuronal type.

Another way to improve connectivity estimates is to use stimulus information. For example, increasing the firing rate can improve quality (Eq 19 and Fig 7), up to a limit. If the firing rate is too high, it becomes harder to infer spikes from fluorescence. A more sophisticated spatio-temporal stimulus scheme can potentially lead to significant improvements in estimation quality [56]. The type of stimulus used can also affect performance. Sensory stimulation usually affects the measured network indirectly, potentially through many layers of neuronal processing. This may result in undesirable common input (“noise correlations”). Optogenetic stimulation does not have this problem, since it stimulates neurons directly by using light sensitive ion channels. However, this type of optical stimulation can potentially interfere with optical recording. Such cross-talk can be minimized by using persistent ion channels [57] (which require only a brief optical stimulus to be activated), or more sophisticated types of stimulation schemes [58, 59]. Such optogenetic approaches, coupled with the inference and experimental design methods described here, have the potential to lead to significantly improved connectivity estimates.

Even if all the neuronal inputs are eventually observed, if the observation probability *p*_obs_ is low then the variance due to the unobserved inputs may still be high, since, at any given time, most of the inputs to each neuron will be unobserved (see also [28]). As a result, the duration of the experiment required for accurate inference increases quadratically with the inverse of the observation probability (Eqs (18)–(19) and Fig 7), and weak weights become much harder to infer (Fig 6). Note this variance may be significantly reduced if we only aim to infer the input connections to only a few neurons (Fig 8). However, in many cases we wish to infer the entire network. In those cases the variance issue will persist, for any fixed observation strategy that does not take into account any prior information on the network connectivity.

However, there might be a significant improvement in performance if we can focus the observations on synaptic connections which are more probable. This way, we can effectively reduce input noise from unobserved neurons, and improve the signal to noise ratio. As a simple example, suppose we know the network is divided into several disconnected components. In this case, we should scan each sub-network separately, *i.e*., there is no point in interleaving spike observations from two disconnected sub-networks. How should one focus observations in the more general case, making use of past observations in an online manner? Again, we leave this “active learning” problem as an important direction for future research.

### 11 Conclusions

In this work we suggest a “shotgun” experimental design, in which we infer the connectivity of a neural network from highly sub-sampled spike data. This is done in order to overcome experimental limitations stemming from the bounded scanning speed of any imaging device.

To do this, we develop a statistical expected loglikelihood-based Bayesian method. This method formally captures the intuitive notion that empiric spike correlations and mean spike rates are approximately the sufficient statistics for connectivity inference. Exploiting these sufficient statistics, our method has two major advantages over previous related approaches: (1) it is orders of magnitude faster (2) it can be used even when the spike data is massively sub-sampled.

We show that by using a double serial scanning scheme, all spike rates and correlations can be eventually inferred (and therefore neural connectivity). We demonstrate numerically that our method works efficiently in a simulated model with highly sub-sampled data and thousands of neurons. We conclude that the limited scanning speed of an imaging device recording neuronal activity is not a fundamental barrier which prevents consistent estimation of network connectivity.

## Supporting Information

S1 Text Technical appendix with full mathematical derivations and algorithmic details. (PDF)
