# Correction: Efficient "Shotgun" Inference of Neural Connectivity from Highly Sub-sampled Activity Data

**DOI:** 10.1371/journal.pcbi.1004657

**Published:** 2015-12-03

**Authors:** The PLOS Computational Biology Staff

An error was introduced during the typesetting process. In the fourth line of Equation 13, there should be no dot above the ‘*T*’ to the right of the approximate equality sign. The corrected line should be:

$$\overset{\left(1\right)}{\approx }T\sum_{i=1}^{N}\left[{\langle S_{i,t}U_{i,t}\rangle }_{T}-\int \;\text{ln}\left(1+e^{x}\right)N\left(x|{\langle U_{i,t}\rangle }_{T},{\text{Var}}_{T}\left(U_{i,t}\right)\right)dx\right]$$

The publisher apologizes for this error.
