# Supplementary material for: Efficient "Shotgun" Inference of Neural Connectivity from Highly Sub-sampled Activity Data
Source: PLoS Comput Biol. 2015 Oct 14;11(10):e1004464. doi: 10.1371/journal.pcbi.1004464 (PMC4605541; doi:10.1371/journal.pcbi.1004464)
Supplement: S1 Text — (PDF) [file pcbi.1004464.s001.pdf]

Efficient "shotgun" inference of neural connectivity from highly sub-sampled activity data

Daniel Soudry*[1], Suraj Keshri[2], Patrick Stinson[1], Min-hwan Oh[2], Garud Iyengar[2], Liam Paninski[1]
1 Department of Statistics, Department of Neuroscience, the Center for Theoretical Neuroscience, the Grossman Center for the Statistics of Mind, the Kavli Institute for Brain Science, and the NeuroTechnology Center, Columbia University, New York, NY, USA
2 Department of Industrial Engineering and Operations Research, Columbia University, New York, NY, USA
∗ **E-mail: daniel.soudry@gmail.com**

Supporting Information - Text S1

A Likelihood Properties

We examine the profile loglikelihood in Eq. 8, divided by T for convenience:

$$\frac{1}{T}\max_{\mathbf{b}}\ln P\left(\mathbf{S}|\mathbf{W},\mathbf{b}\right)\approx\sum_{i=1}^{N}\left[\sum_{j=1}^{N}\left[W_{i,j}\Sigma_{i,j}^{(1)}\right]-h\left(m_{i}\right)\sqrt{1+\frac{\pi}{8}\sum_{k,j}W_{i,j}\Sigma_{k,j}^{(0)}W_{i,k}}\right].$$

Note this expression is a sum over N separable components corresponding to rows of the $\mathbf{W}$ matrix (*i.e.*, all the inputs a single neurons receives). Each component can be maximized separately, as long as we also use similarly separable log-priors (like the L1 penalty we use - Eq. 46 below). We exploit this to reduce the number of indices in the following derivations, by examining only the profile loglikelihood of a single row:

$$L_{i}\triangleq\mathbf{\Sigma}_{i,\cdot}^{(1)}\mathbf{W}_{i,\cdot}^{\top}-h\left(m_{i}\right)\sqrt{1+\frac{\pi}{8}\mathbf{W}_{i,\cdot}\mathbf{\Sigma}^{(0)}\mathbf{W}_{i,\cdot}^{\top}}\,. \tag{20}$$

A.1 Gradient, Hessian and Concavity

It straightforward to find the gradient of the approximated profile loglikelihood L_i. Clearly, $\nabla_{\mathbf{W}_{i,\cdot}}L_j=0$, if $i\neq j$. Otherwise,

$$\nabla_{\mathbf{W}_{i,\cdot}}L_{i} = \mathbf{\Sigma}_{i,\cdot}^{(1)}-\frac{\pi}{8}h\left(m_{i}\right)\frac{\mathbf{W}_{i,\cdot}\mathbf{\Sigma}^{(0)}}{\sqrt{1+\frac{\pi}{8}\mathbf{W}_{i,\cdot}\mathbf{\Sigma}^{(0)}\mathbf{W}_{i,\cdot}^{\top}}}\,. \tag{21}$$

Next, to obtain the Hessian, we take another derivative

$$\begin{aligned}\nabla_{\mathbf{W}_{i,\cdot}}\left(\nabla_{\mathbf{W}_{j,\cdot}^{\top}}L_{i}\right) &= \frac{\frac{\pi}{8}h\left(m_{i}\right)\delta_{i,j}}{\sqrt{1+\frac{\pi}{8}\mathbf{W}_{i,\cdot}\mathbf{\Sigma}^{(0)}\mathbf{W}_{i,\cdot}^{\top}}} \\ &\cdot \left(\frac{\pi}{8}\mathbf{\Sigma}^{(0)}\mathbf{W}_{j,\cdot}^{\top}\mathbf{W}_{i,\cdot}\mathbf{\Sigma}^{(0)}-\left(1+\frac{\pi}{8}\mathbf{W}_{i,\cdot}\mathbf{\Sigma}^{(0)}\mathbf{W}_{j,\cdot}^{\top}\right)\mathbf{\Sigma}^{(0)}\right).\end{aligned} \tag{22}$$

Next, we show that L_i is concave. To do this, we need to prove that for any $\mathbf{W}$ and any vector $\mathbf{z}\in\mathbb{R}^{N\times1}$

$$\mathbf{z}^{\top}\left(\nabla_{\mathbf{W}_{i,\cdot}}\left(\nabla_{\mathbf{W}_{j,\cdot}}L_{i}\right)\right)\mathbf{z}\leq0\,.$$

Therefore, we need to calculate the sign of

$$\frac{\pi}{8}\left(\mathbf{W}_{i,\cdot}^{\top}\mathbf{\Sigma}^{(0)}\mathbf{z}\right)^{2}-\left(1+\frac{\pi}{8}\mathbf{W}_{i,\cdot}^{\top}\mathbf{\Sigma}^{(0)}\mathbf{W}\right)\mathbf{z}^{\top}\mathbf{\Sigma}^{(0)}\mathbf{z}$$

Since (given T is large enough) $\mathbf{\Sigma}^{(0)}$ is positive definite (since the expectation of $\mathbf{\Sigma}^{(0)}$ is a covariance matrix), we can decompose $\mathbf{\Sigma}^{(0)}=\mathbf{\Lambda}\mathbf{\Lambda}^{\top}$. Denoting $\boldsymbol{u}\triangleq\mathbf{\Lambda}^{\top}\mathbf{W}$ and $\mathbf{v}\triangleq\mathbf{\Lambda}^{\top}\mathbf{z}$ we find that the last line can be written as

$$\frac{\pi}{8}\left(\left(\boldsymbol{u}^{\top}\boldsymbol{v}\right)^{2}-\boldsymbol{u}^{\top}\boldsymbol{u}\boldsymbol{v}^{\top}\boldsymbol{v}\right)-\boldsymbol{v}^{\top}\boldsymbol{v}\overset{(1)}{\leq}-\boldsymbol{v}^{\top}\boldsymbol{v}\overset{(2)}{\leq}0\,,$$

where in (1) we used the Cauchy-Schwarz inequality $\left(\boldsymbol{u}^{\top}\boldsymbol{v}\right)^{2}\leq\boldsymbol{u}^{\top}\boldsymbol{u}\boldsymbol{v}^{\top}\boldsymbol{v}$, and in (2) we used the fact that $\boldsymbol{v}^{\top}\boldsymbol{v}\geq 0$ for any vector $\boldsymbol{v}$. Therefore, L_i is concave in every row separately, and so jointly in all rows.

A.2 Maximum Likelihood Estimator

We wish to find $\mathbf{W}$ that solves

$$0=\nabla_{\mathbf{W}_{i,\cdot}}\max_{\mathbf{b}}\ln P\left(\mathbf{S}|\mathbf{W},\mathbf{b}\right)\,.$$

Using Eq. 21, we obtain

$$0=\mathbf{\Sigma}_{i,\cdot}^{(1)}-\frac{\pi}{8}h\left(m_i\right)\frac{\mathbf{W}_{i,\cdot}\mathbf{\Sigma}^{(0)}}{\sqrt{1+\frac{\pi}{8}\mathbf{W}_{i,\cdot}\mathbf{\Sigma}^{(0)}\mathbf{W}_{i,\cdot}^{\top}}}\,. \tag{23}$$

We define

$$a_i\triangleq\frac{\frac{\pi}{8}h\left(m_i\right)}{\sqrt{1+\frac{\pi}{8}\mathbf{W}_{i,\cdot}\mathbf{\Sigma}^{(0)}\mathbf{W}_{i,\cdot}^{\top}}} \tag{24}$$

so we can write Eq. 23 as

$$0=\mathbf{\Sigma}_{i,\cdot}^{(1)}-a_i\mathbf{W}_{i,\cdot}\mathbf{\Sigma}^{(0)}\,,$$

which is solved by

$$\mathbf{W}_{i,\cdot}=\frac{1}{a_i}\mathbf{\Sigma}_{i,\cdot}^{(1)}\left(\mathbf{\Sigma}^{(0)}\right)^{-1}\,, \tag{25}$$

Substituting this into Eq. 24, we have

$$\sqrt{1+\frac{\pi}{8a_i^2}\mathbf{\Sigma}_{i,\cdot}^{(1)}\left(\mathbf{\Sigma}^{(0)}\right)^{-1}\left(\mathbf{\Sigma}_{i,\cdot}^{(1)}\right)^{\top}}=\frac{\frac{\pi}{8}h\left(m_i\right)}{a_i}$$

so

$$a_i=\sqrt{\left(\frac{\pi}{8}h\left(m_i\right)\right)^{2}-\frac{\pi}{8}\mathbf{\Sigma}_{i,\cdot}^{(1)}\left(\mathbf{\Sigma}^{(0)}\right)^{-1}\left(\mathbf{\Sigma}_{i,\cdot}^{(1)}\right)^{\top}}\,. \tag{26}$$

In conclusion, the ML estimator of $\mathbf{W}_{i,\cdot}$ is given by Eq. 25, where a_i is given by Eq. 26. This estimate would coincide with any MAP estimate when $T\to\infty$. Note that a finite solution exists only if the last expression has a real value.

Interestingly, this ML estimate is a re-scaled version of the ML estimate in a simple linear Gaussian neuron model (in that case $a_i=0.5$). Though one can obtain a Gaussian model also by assuming that the neural input is small (*e.g.*, due to weak weights), it will not have the same scale a_i. Specifically, if

the input is small we can linearize the profile loglikelihood (Eq. 8), and reduce it to the simple quadratic form

$$\max_{\mathbf{b}} \ln P\left(\mathbf{S}|\mathbf{W},\mathbf{b}\right) \approx T\sum_{i=1}^{N}\left[\sum_{j=1}^{N}\left[W_{i,j}\Sigma^{(1)}_{i,j}\right] - h\left(m_i\right)\frac{\pi}{16}\sum_{k,j}W_{i,j}\Sigma^{(0)}_{k,j}W_{i,k}\right] . \tag{27}$$

This is similar to the loglikelihood that we would have a obtained if we had assumed a linear Gaussian neuron model, albeit with different constants. In this linearized case (27), the ML estimate will have same form as in Eq. 25, though with $a_i = \frac{\pi}{8}h\left(m_i\right)$. Therefore, when the neuronal input is strong, the non-linear nature of the neurons only modifies the gain constant a_i.

An identical ML estimate was obtained, using similar approximations, for a Poisson neuron model [1,2], albeit with a different re-scaling. This result suggests a generality of the form of the ML estimate under the ELL approximation. This form results from the Gaussian approximation (CLT) that was used on the summed neuronal input, as we explain next.

A.3 Alternative neuron models

Next, we will show that, under rather mild assumptions, Eq. 25 will hold for other spiking neuron models. Only the constant a_i will depend on the specific neuron model. For the logistic model, we have Eqs. 1 and 2. In the general case,

$$P\left(S_{i,t}=1|U_{i,t}\right) = f\left(U_{i,t}\right) ,$$

where $f\left(\cdot\right)$ is some function (not necessarily a logistic. The only restriction we impose on $f\left(\cdot\right)$ is that $f\left(\cdot\right) - 0.5$ is an odd function, so that

$$P\left(S_{i,t}=0|U_{i,t}\right) = 1 - f\left(U_{i,t}\right) = f\left(-U_{i,t}\right) ,$$

We examine the expected loglikelihood in this general case. For simplicity, in all derivations we will again assume $\mathbf{G}=0$. We define $\left\langle X_t|Y_t=y\right\rangle_T$ as the conditional empirical mean over X_t, given $Y_t=y$ and $\mathrm{Var}_T\left(X_t|Y_t=y\right)$ as the conditional empirical variance. We find that

$$\begin{aligned} & \frac{1}{T}\log P\left(\mathbf{S}|\mathbf{W},\mathbf{b}\right) \\ = \; & \left\langle S_{i,t}\right\rangle \left\langle \log f\left(U_{i,t}\right)|S_{i,t}=1\right\rangle_T + \left(1-\left\langle S_{i,t}\right\rangle\right)\left\langle \log\left(f\left(-U_{i,t}\right)\right)|S_{i,t}=0\right\rangle_T \\ \approx \; & \left\langle S_{i,t}\right\rangle g\left(\mu_i^+,\sigma_i^2\right) + \left(1-\left\langle S_{i,t}\right\rangle\right) g\left(\mu_i^-,\sigma_i^2\right) , \end{aligned}$$

where, in the last line, we used the CLT approximation, denoted

$$g\left(x,y\right) \triangleq \int_{-\infty}^{\infty}\log f\left(u\right)\mathcal{N}\left(u|x,y\right)du \tag{28}$$

$$\mu_i^+ \triangleq \left\langle U_{i,t}|S_{i,t}=1\right\rangle = \mathbf{W}_{i,\cdot}\left\langle \mathbf{S}_{\cdot,t-1}|S_{i,t}=1\right\rangle + b_i \tag{29}$$

$$\mu_i^- \triangleq -\left\langle U_{i,t}|S_{i,t}=0\right\rangle = -\mathbf{W}_{i,\cdot}\left\langle \mathbf{S}_{\cdot,t-1}|S_{i,t}=0\right\rangle - b_i , \tag{30}$$

and also approximated

$$\sigma_i^2 \triangleq \mathrm{Var}_T\left(U_{i,t}|S_{i,t}=0\right) \approx \mathrm{Var}_T\left(U_{i,t}|S_{i,t}=1\right) \approx \mathrm{Var}_T\left(U_{i,t}\right) = \mathbf{W}_{i,\cdot}\mathbf{\Sigma}^{(0)}\mathbf{W}_{i,\cdot}^{\top} . \tag{31}$$

Defining

$$g_{x,i}^{\pm} = \left.\frac{\partial}{\partial x}g\left(x,\sigma_i^2\right)\right|_{x=\mu_i^{\pm}} ; \; g_{y,i}^{\pm} = \left.\frac{\partial}{\partial y}g\left(\mu_i^{\pm},y\right)\right|_{y=\sigma_i^2}$$

differentiating Eq. 30 and equating to zero, we obtain

$$0 = \frac{1}{T}\nabla_{b_i}\log P\left(\mathbf{S}|\mathbf{W},\mathbf{b}\right) = \left\langle S_{i,t}\right\rangle g_{x,i}^{+} + \left(1-\left\langle S_{i,t}\right\rangle\right) g_{x,i}^{-} \tag{32}$$

and

$$
\begin{aligned}
0 &= \frac{1}{T}\nabla_{\mathbf{W}_{i,\cdot}}\log P\left(\mathbf{S}|\mathbf{W},\mathbf{b}\right)\\
&= \left\langle S_{i,t}\right\rangle \left(g_{x,i}^{+}\left\langle \mathbf{S}_{\cdot,t-1}|S_{i,t}=1\right\rangle +2g_{y,i}^{+}\boldsymbol{\Sigma}^{(0)}\mathbf{W}_{i,\cdot}^{\top}\right)+\left(1-\left\langle S_{i,t}\right\rangle \right)\left(g_{x,i}^{-}\left\langle \mathbf{S}_{\cdot,t-1}|S_{i,t}=0\right\rangle +2g_{y,i}^{-}\boldsymbol{\Sigma}^{(0)}\mathbf{W}_{i,\cdot}^{\top}\right)\\
&= \left\langle S_{i,t}\right\rangle g_{x,i}^{+}\left(\left\langle \mathbf{S}_{\cdot,t-1}|S_{i,t}=1\right\rangle -\left\langle \mathbf{S}_{\cdot,t-1}|S_{i,t}=0\right\rangle \right)+2\left(\left\langle S_{i,t}\right\rangle g_{y,i}^{+}+\left(1-\left\langle S_{i,t}\right\rangle \right)g_{y,i}^{-}\right)\boldsymbol{\Sigma}^{(0)}\mathbf{W}_{i,\cdot}^{\top}\,, \qquad (33)
\end{aligned}
$$

where in the last line used Eq. 32 to simplify the result. Defining

$$
a_{i}=2\frac{\left\langle S_{i,t}\right\rangle g_{y,i}^{+}+\left(1-\left\langle S_{i,t}\right\rangle \right)g_{y,i}^{-}}{g_{x,i}^{+}}\left(1-\left\langle S_{i,t}\right\rangle \right), \qquad (34)
$$

we obtain

$$
\begin{aligned}
\mathbf{W}_{i,\cdot} &= \frac{\left\langle S_{i,t}\right\rangle \left(1-\left\langle S_{i,t}\right\rangle \right)}{a_{i}}\left[\left\langle \mathbf{S}_{\cdot,t-1}|S_{i,t}=1\right\rangle -\left\langle \mathbf{S}_{\cdot,t-1}|S_{i,t}=0\right\rangle \right]^{\top}\left(\boldsymbol{\Sigma}^{(0)}\right)^{-1} \qquad (35)\\
&= \frac{1}{a_{i}}\boldsymbol{\Sigma}_{i,\cdot}^{(1)}\left(\boldsymbol{\Sigma}^{(0)}\right)^{-1}, \qquad (36)
\end{aligned}
$$

where in the last line we used the identity

$$
\begin{aligned}
\left(\boldsymbol{\Sigma}_{i,\cdot}^{(1)}\right)^{\top} &= \left\langle S_{i,t}\mathbf{S}_{\cdot,t-1}\right\rangle -\left\langle S_{i,t}\right\rangle \left\langle \mathbf{S}_{\cdot,t}\right\rangle \\
&= \left\langle S_{i,t}\right\rangle \left\langle \mathbf{S}_{\cdot,t-1}|S_{i,t}=1\right\rangle -\left\langle S_{i,t}\right\rangle \left(\left\langle S_{i,t}\right\rangle \left\langle \mathbf{S}_{\cdot,t-1}|S_{i,t}=1\right\rangle -\left(1-\left\langle S_{i,t}\right\rangle \right)\left\langle \mathbf{S}_{\cdot,t-1}|S_{i,t}=0\right\rangle \right)\\
&= \left\langle S_{i,t}\right\rangle \left(1-\left\langle S_{i,t}\right\rangle \right)\left(\left\langle \mathbf{S}_{\cdot,t-1}|S_{i,t}=1\right\rangle -\left\langle \mathbf{S}_{\cdot,t-1}|S_{i,t}=0\right\rangle \right)
\end{aligned}
$$

In conclusion, in Eq. 36 we get the same ML estimate, as in the case of a logistic neuron model (Eq. 25), up to a change in the proportionality constant a_i. This constant can be found by substituting Eq. 34 into Eq. 35 and solving the resulting equation. Note, however, the following:

1. The MAP estimate can change between neuron models, due to the addition of the log prior.

2. Here, we assumed the input current is Gaussian *given* the output spikes (in Eq. 30), and that the input current variance is independent of the output spikes (Eq. 31), while in the logistic model, we assumed only that the input current is Gaussian (without conditioning on the input).

3. This generic result for the ML estimate (Eq. 35) can be explained geometrically. This weight vector is normal to the decision hyperplane (a classification rule for whether or not an output spike happened given the input spikes) that would be obtained by Linear Discriminant Analysis (LDA, [3]). This LDA decision hyperplane is the Bayes optimal decision rule given (wrong) assumption that the input spikes are Gaussian given the output spikes, and that the input spikes' $\mathbf{S}_{\cdot,t-1}$ covariance does not depend on the output spikes $S_{i,t}$. Here, we have the same assumptions (in Eqs. 30 and 31), but on the input current $U_{i,t}$, and not on the input spikes $\mathbf{S}_{\cdot,t-1}$. However, nothing in our derivation would change if we use LDA's stricter assumptions instead. Therefore, we get the same solution as LDA.

B Simulation details

In this section we provide extensive details behind the numerical simulations. The code is available on github: `https://github.com/danielso/Shotgun`. All simulations were done using Matlab 2013b on an Intel i7-4500 laptop, with a Windows 8.1 operating system.

B.1 Network model simulation

We test the shotgun scheme on simulated spike data from a recurrent GLM-based spiking neural network. In sections 5 and 6 we model the simulated network with biologically plausible parameters from the mouse visual cortex. Here we describe those parameters and how they were chosen.

- Each time bin corresponds to 10 ms, which is compatible with the timescales of neuronal integration [4].

- In the mouse visual cortex, neuronal density is about $\rho = 10^{-4}\,[\mu m]^{-3}$ [5]. Consequently, if we image neural tissue from a volume of size V, we should expect to record, at most, from $N = \rho V$ neurons. We assume that all neurons from the volume are indeed captured (*e.g.*, as in [6], where 411 neurons are recorded from a $200 \times 200 \times 100\mu m$ volume) and that these neurons' locations (centers of mass) are randomly distributed in the imaged volume. Specifically, to construct a network of size N, we uniformly sample N neuronal locations in a cube $[0, L]^3$ where $L^3 = N/\rho$.

- Following [7, Figure 1E], we determine the connection probability (the probability that $W_{i,j}$ is not zero) according to
$$f(d) = p_0 \exp\left(-\frac{d}{d_0}\right) \tag{37}$$
where $p_0 = 0.2$, $d_0 = 200\mu m$ is the typical length scale, and d is the Euclidean distance between two neurons. Empirically, this gives an average connection probability (between any two neurons) near $p_{\mathrm{conn}} \approx 0.1$, which is in the range estimated by [5, 7].

- All outgoing weights from a neuron have the same sign, following Dale's law. 20% of the neurons are inhibitory, while 80% are excitatory.

- The mean firing rate was set to 5Hz (*i.e.,* 0.05 probability of a spike in each time bin), similarly to cells receiving sensory input (for simplicity, we assume a constant signal). Specifically, since inhibitory neurons typically fire faster, the mean firing rate of the excitatory and inhibitory neurons was set to 4.5Hz and 7.5Hz, respectively. Firing rates were tuned by adjusting the biases $\mathbf{b}$ automatically using the stochastic approximation method [8]. The resulting distribution of b_i typically had a mean near -2.9 and variance near 0.16. To interpret these values, recall the logistic spiking model (Eq. 1). Near these negative values, perturbing b_i by Δ approximately results in the mean spiking probability of neuron i being multiplied by $\exp(\Delta)$, up to some saturation level starting near 0.5. This exponential increase can be also observed experimentally, in [9, Fig 2C].

- The distribution of the excitatory synaptic strengths is known to be heavy-tailed, and typically described as log-normal over approximately 2.5 decades as in [10] and [9]. Therefore, as in [9], we sampled the excitatory weights from a log-normal distribution in which the mean is similar to the standard deviation. The distribution of the inhibitory weights is much harder to measure experimentally, and therefore is not known, to the best of our knowledge. Therefore, as in [9], the inhibitory weights were sampled from Gaussian distribution with the same variance as the excitatory weights. Inhibitory synapses are expected to have a greater effect on the spike generation process, since inhibitory synapses tend to be located on or near the soma of the nerve cell, whereas excitatory ones are most abundant on dendrites [11]. Thus, the mean of the inhibitory weight was set about four times stronger than that of the excitatory weights. This value promotes an excitation-inhibition balance [12, 13], in accordance with the conditions on the weight ratios described in [12] were met. Specifically, for inhibitory to excitatory weights we used a mean of 1, for inhibitory to inhibitory weights we used a mean of 0.8, for excitatory to excitatory weights we used a mean of 0.25, and for excitatory to inhibitory weights we used a mean of 0.2. To avoid unrealistically low or high weight values (so we get a range of 2.5 decades), we introduced a lower bound at 0.01 and an

upper bound at 3. Any weights crossing these bounds were re-sampled. For self connectivity, we used $W_{i,i} \sim \mathcal{N}(-2, 0.2)$ to account for the refractory period (which is typically stronger than any synapse).

- In the resulting model, the distribution of the excitatory weights (Fig S1 B) has a mode near 0.11, so most synapses' activations contribute an input which is about 30 times smaller than the "spiking threshold" ($-b_i \approx 2.9$). Therefore, for most synapses, a few dozen pre-synaptic spikes are typically required in order to generate a post-synaptic spike. This is similar to what is observed experimentally in [9, Fig 2C], in which most synapses (near the 0.1ns conductivity mode of [9, Fig 1A]) increase spike probability approximately 0.03 above chance level. This indicates that the weight magnitudes are in a physiologically reasonable range. Furthermore, the resulting model exhibits firing patterns similar to those observed in biological neural networks (Fig S1 A). Specifically, the distribution of spike correlations between neurons (Fig S1 C) and the distribution of population firing rates (Fig S1 D), are comparable to those observed in experimental data [14]. Note that we are being somewhat conservative here (assuming a worst-case scenario). This because the firing rate in [14] ranged from 0.3Hz to 4.5Hz, while the excitatory cells in our model fire at 4.5Hz. Therefore, at the same firing rates, our model will probably have somewhat lower correlations than in realistic data (which makes inference harder in our model). For example, using a firing rate of 1Hz instead, somewhat decreases the correlations magnitude (Fig S1C) to a smaller range ($[-0.03, 0.11]$, not shown).

- We used a double-serial observation scheme: the network was scanned serially using two devices in contiguous blocks with incommensurate periods, as described in section 3.5 (and Fig 1, I,J). The dwell time of both devices was 1 sec and π sec, respectively. These specific values were chosen to allow spike inference from the calcium traces.

- The rest of the parameters are individually set for each figure - N, the number of observed neurons, T, the observation times and p_{obs}, the mean fraction of neurons observed at each time bin, *i.e.*, the empiric observation probability in the shotgun scheme.

- To demonstrate robustness, in every simulation we always add another $0.2N$ neurons which are never observed (as in [15]). Though this number is rather arbitrary, our numerical results hold even it is somewhat increased (not shown). However, if the number of unobserved neurons is significantly larger than the number of observed neurons, we would be required to incorporate latent variables in the model, as in [16].

B.2 Single neuron simulation

In the network simulations, we examine networks with up to $N = 2048$ observed neurons, which are relatively easy to simulate on a single machine. Such network sizes are often used in theoretical studies. However, in these networks, each neuron has up to a few hundred inputs with non-zero weights, which is at least an order of magnitude less then the real numbers. To verify that our results hold for a more realistic number of inputs, we will also examine a simulation of a single neuron with many inputs. A single neuron can be much easier to simulate than a whole network, if we exploit the fact that our estimation method depends only on the approximate sufficient statistics $(\mathbf{m}, \mathbf{\Sigma}^{(k)})$.

Specifically, in our model an "output neuron" is observed together with $N = 10626$ input neurons, 968 of which have non-zero weights to the output neuron. An additional 152 inputs to the neuron (with non-zero weights) are never observed. In order to make sure that the input spikes have the correct statistics, we still need to run a network simulation. However, in order to avoid simulating

the full network with $N = 10626$ neurons, we only simulate the spikes for the inputs with the non-zero weights, together with additional 200 inputs that have zero weights. In total the simulated network has only $N = 1321$ neurons - 1320 input neurons which are connected to each other as in our network model (section B.1), and one output neuron connected to all the other neurons (using the same weight distribution), except 200.

These 200 (zero weight) input neurons are then used to imitate an additional 9306 (zero weight) input neurons that were not simulated. To do this, we sample (uniformly, with replacement) from the spike statistics $(\mathbf{m}, \mathbf{\Sigma}^{(k)})$ of these 200 neurons (together with output neuron) to generate the same statistics for the zero weight input neurons that were not simulated. Thus, with all the spikes statistics $(\mathbf{m}, \mathbf{\Sigma}^{(k)})$ in hand, we estimate the connectivity matrix $\mathbf{W}$.

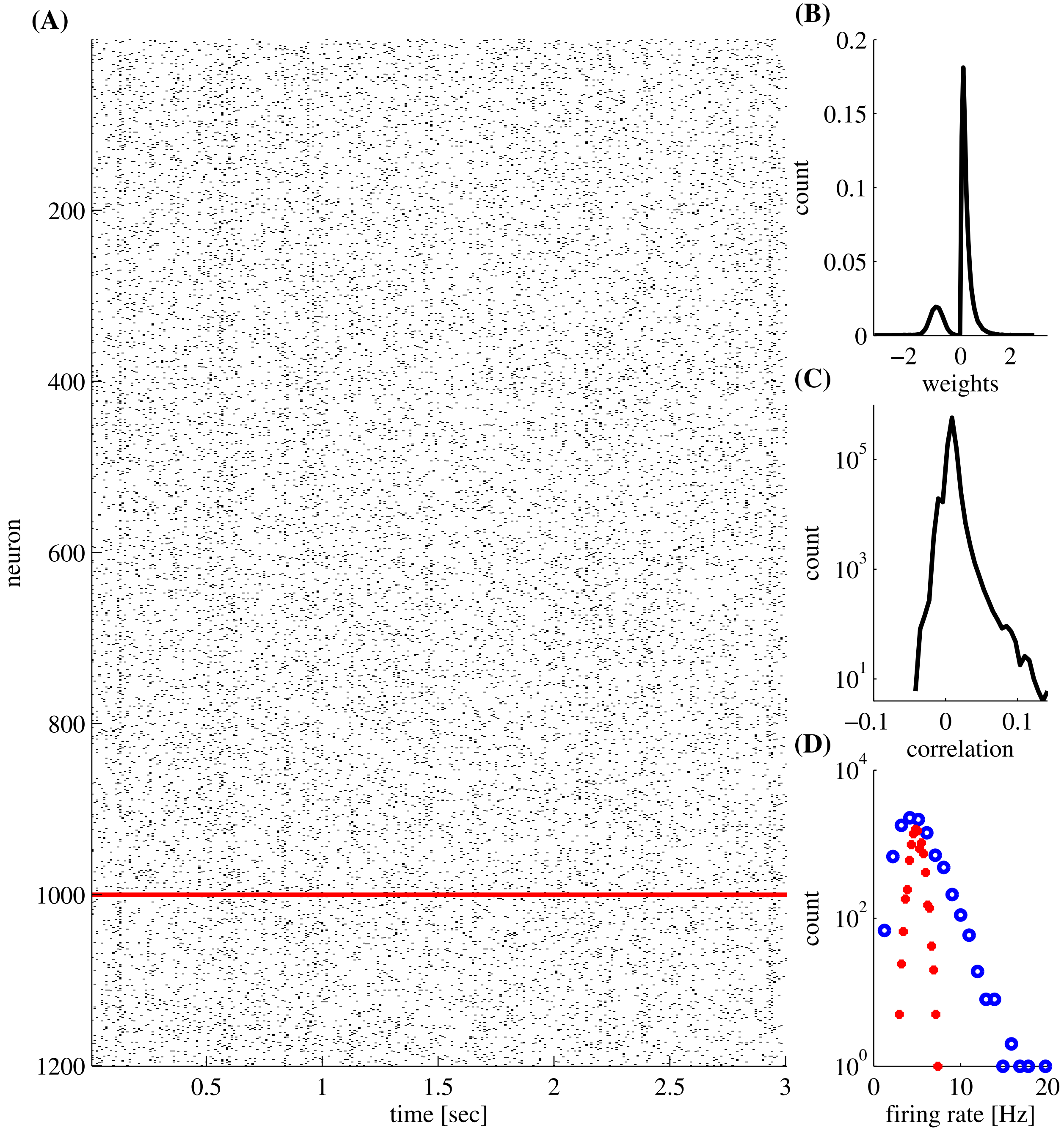

Figure S1. Firing patterns in simulated network with $N = 1000$. (A) spike raster plot snapshot. The 200 cells below red line are never observed. The 1000 cells above red line are observed using a double serial scanning scheme (Fig 1, I,J). The mean firing rate is 4.5Hz for the excitatory neurons, and 7.5Hz for the inhibitory neurons. **(B)** Histogram of nonzero weights - excitatory weights have a broad (log-normal) distribution. **(C)** Histogram of the spike correlation values, ρ, for all pairs of different neurons. The correlation ρ was calculated as in [14], using 20ms timebins. The distribution of ρ is comparable to that of real spike data [14, Fig 1d], where most correlations are weak, *i.e.* $|\rho| < 0.1$. **(D)** Distribution of the mean population firing rates (blue circles), *i.e.*, the fraction of the cells spiking in a time bin, divided by the time bin duration (10 msec). For comparison, we plot the same distribution after the time bins have been randomly shuffled for each neuron (red asterisks). We see that population "bursts" are significantly more common than chance level, similarly to real data [14, Fig 1e].

B.3 Fluorescence observations and spike inference - implementation details

In section 7 we add another layer of complexity and infer connectivity from fluorescence traces. Here we describe the fluorescence model and how the spikes are inferred from fluorescence. We use the same network model as in section B.1, with $N = 50$ observed neurons and the length of the experiment $T = 5.5$ hours. The frame rate here is 100Hz (i.e., the time bin $dt = 10$ msec). The only difference in the network is that we decreased the mean spontaneous firing rates (to 3Hz for excitatory neurons, 4.5Hz for inhibitory neurons), simplifying spike inference. However, the decrease in the firing rate also effectively reduces the amount of available data, and consequently, the quality of the connectivity inference (see Fig 7).

We assume that the observed fluorescence traces are a noisy, low pass filtered version of the spikes (similarly to [17] and references therein), *i.e.* for each neuron i:

$$C_{t+1,i} = \gamma C_{t,i} + S_{t,i} \tag{38}$$

$$Y_{t,i} = C_{t,i} + \epsilon_{t,i}\,, \tag{39}$$

where $C_{t,i}$ is the internal calcium concentration at time bin t, $Y_{t,i}$ is the observed fluorescence, $\epsilon_{t,i}$ a Gaussian white observation noise with zero mean and standard deviation of 0.2 or 0.4 (these values were chosen to obtain typically noisy traces in Fig 10A), and $\gamma = 0.97$, which determines the time constant. For a time bin of $dt = 10\,\text{ms}$, as we use here, the timescale of fluorescence decay is $\tau = \frac{dt}{1-\gamma} = 300\,\text{msec}$. This is similar to the decay timescale of $\tau = 285.4\,\text{msec}$ of the GCaMP6f calcium fluorescence indicator, which we estimated from publicly available data [18] using the code in [17].

From the fluorescence traces, we infer the underlying spikes using a simple greedy "peeling" algorithm, similar to [19]. Briefly, we first convolve the trace with a low pass filter, matched to the filter of the slow calcium dynamics (Eqs. 38). The filter parameters are estimated from the simulated fluorescence traces using the code in [17]. Then we iteratively repeat the following steps: (1) identify a spike as the maximal peak of this trace, then (2) subtract from this trace the identified spike, convolved with the matched filter. We stop this process when there are no more peaks that, when subtracted, reduce the overall variance.

B.4 Quality Measures

We use four main measures to assess the quality of the estimated matrix $\hat{\mathbf{W}}$ in comparison to the "ground truth" matrix $\mathbf{W}$. First, we define

$$\langle\langle W \rangle\rangle \triangleq \frac{1}{N^2}\sum_{i,j} W_{i,j}\,.$$

The measures we use are:

- The square root of the coefficient of determination (R^2):

$$R \triangleq \sqrt{1 - \frac{\sum_{i,j}\left(W_{i,j} - \hat{W}\right)^2}{\sum_{i,j}\left(W_{i,j} - \langle\langle W \rangle\rangle\right)^2}} \tag{40}$$

- Correlation:

$$C \triangleq \frac{\sum_{i,j}(W_{i,j} - \langle\langle W \rangle\rangle)(\hat{W}_{i,j} - \langle\langle \hat{W} \rangle\rangle)}{\sqrt{\sum_{i,j}(W_{i,j} - \langle\langle W \rangle\rangle)^2 \sum_{i,j}(\hat{W}_{i,j} - \langle\langle \hat{W} \rangle\rangle)^2}} \tag{41}$$

- Zero matching:

$$Z \triangleq 1 - \frac{1}{2}\frac{\sum_{i,j}\left(\mathcal{I}(W_{i,j} = 0)\mathcal{I}(\hat{W}_{i,j} \neq 0) + \mathcal{I}(W_{i,j} \neq 0)\mathcal{I}(\hat{W}_{i,j} = 0)\right)}{\sum_{i,j}\mathcal{I}(W_{i,j} = 0)} \tag{42}$$

- Sign matching:

$$S \triangleq 1 - \frac{1}{2}\frac{\sum_{i,j}\left|\text{sign}(W_{i,j}) - \text{sign}(\hat{W}_{i,j})\right|\mathcal{I}(W_{i,j} \neq 0)\mathcal{I}(\hat{W}_{i,j} \neq 0)}{\sum_{i,j}\mathcal{I}(W_{i,j} \neq 0)\mathcal{I}(\hat{W}_{i,j} \neq 0)} \tag{43}$$

High values indicate high quality of estimation, with 1 indicating zero error. To simplify the presentation scale, if a measure becomes negative or imaginary, we set it to zero.

The sign and zero matching measures depend on the choice of regularization parameter λ in the $L1$ penalty, which was adjusted obtain the expected level of sparsity. Also, the sign measure captures only those connections in which both the truth and the estimate are non-zero, which can be arbitrarily low. To have a clear understanding of specificity-sensitivity tradeoff in our estimation procedure, for each type of weight (excitatory/inhibitory, denoted by $\pm$), we additionally define the true positive rate (sensitivity)

$$\text{TPR}_{\pm} = \frac{\sum_{i,j}\mathcal{I}(\pm W_{i,j} > 0)\mathcal{I}(\pm\hat{W}_{i,j} > 0)}{\sum_{i,j}\mathcal{I}(\pm W_{i,j} > 0)} \tag{44}$$

and the false positive rate (specificity)

$$\text{FPR}_{\pm} = \frac{\sum_{i,j}\mathcal{I}(\pm W_{i,j} < 0)\mathcal{I}(\pm\hat{W}_{i,j} > 0)}{\sum_{i,j}\mathcal{I}(\pm W_{i,j} < 0)}\,. \tag{45}$$

Using these measures on the inferred weights we draw a Receiver Operating Characteristic (ROC) curve, for each value of λ. The Area Under the Curve (AUC) for the ROC is a popular measure for the quality of the estimator.

C Sparsity inducing L1 prior

As most neurons are not connected, most of the off-diagonal terms in $\mathbf{W}$ are equal to zero, making $\mathbf{W}$ sparse. Taking this prior knowledge into account, we incorporate it into our estimates by placing a Laplace prior on the weights, effectively adding an L_1 norm penalty :

$$\ln P_0(\mathbf{W}) = -\sum_{i,j}\lambda_{i,j}\left|W_{i,j}\right| + C \tag{46}$$

for some set of sparsity parameters $\lambda_{i,j}$. However, the diagonal elements $W_{i,i}$ of the connectivity matrix are typically negative, corresponding to the the cell's own (refractory) post-spike effects. Therefore, we set

$$\lambda_{i,j} = (1 - \delta_{i,j})\,\lambda\,, \tag{47}$$

where $\lambda > 0$. This prior has a number of advantages: the resulting log-posterior of $\mathbf{W}$ (given the full spike train $\mathbf{S}$ and the other system parameters) is a concave function of $\mathbf{W}$, and the maximizer of this posterior, $\hat{\mathbf{W}}_{\text{MAP}}$, is often sparse, *i.e.*, many values of $\hat{\mathbf{W}}_{\text{MAP}}$ are zero. Plugging this prior in the posterior (Eq. 4), together with the simplified profile loglikelihood (Eq. 8), we obtain a LASSO-type problem for $\mathbf{W}$ [20]. By solving this objective we obtain a sparse Maximum A Posteriori (MAP) estimate $\hat{\mathbf{W}}_{\text{MAP}}$. There are many algorithms that can be used to solve such an objective. Usually, the most efficient [21] is the FISTA algorithm [22]; details are given in section C.1. In order to set the value of λ, we assume some rough prior knowledge (*e.g.*, statistics from anatomical data) about the sparsity of $\mathbf{W}$ - *i.e.*, the average number of connections of each neuron in the population. Using this knowledge, it is straightforward to set the value of λ using an exponential search algorithm, as explained in section C.2.

C.1 The FISTA algorithm

We define the proximal operator [21]

$$\left(\mathcal{T}_{\mu}\left(\mathbf{u}\right)\right)_i \triangleq \max\left(1-\frac{\mu}{|u_i|},0\right)u_i\,. \tag{48}$$

FISTA solves the following minimization problem

$$\min_{\mathbf{W}}\left[g\left(\mathbf{W}\right)+\lambda\left\|\mathbf{W}\right\|_1\right]\,.$$

From [22, Eqs. 4.1-4.3], we have the following Algorithm 1, where the components of the gradient $\nabla g\left(\mathbf{W}\right)$ are given by Eq. 21, and the Lipschitz constant l (defined and derived in section C.3) is given by Eq. 49 below.

Algorithm 1 The FISTA algorithm.

Input Initial point $\mathbf{W}^{(k)}$, ∇g and l (Lipschitz constant of ∇g).

Initialize $\mathbf{Y}^{(0)}=\mathbf{X}^{(0)}$, $t_1=1$.

Repeat For $k\geq 1$ compute

$$\begin{aligned}\mathbf{W}^{(k)} &= \mathcal{T}_{\lambda/l}\left[\mathbf{Y}^{(k)}-\frac{1}{l}\nabla g\left(\mathbf{Y}^{(k)}\right)\right]\\ t_{k+1} &= \left(1+\sqrt{1+4t_k^2}\right)/2\\ \mathbf{Y}^{(k+1)} &= \mathbf{W}^{(k)}+\left(\frac{t_k-1}{t_{k+1}}\right)\left(\mathbf{W}^{(k)}-\mathbf{W}^{(k-1)}\right)\,.\end{aligned}$$

C.2 Setting λ using the sparsity constraint

As explained in section C, we use a sparsity promoting prior (Eqs. 46-47), which depends on a regularization constant λ, to generate and estimate $\hat{\mathbf{W}}\left(\lambda\right)$ of the connectivity matrix. Though this constant is unknown in advance, we can set it using the sparsity level of $\hat{\mathbf{W}}\left(\lambda\right)$, defined as

$$\mathrm{spar}\left(\lambda\right)\triangleq\frac{1}{N}\sum_{i,j}\mathcal{I}\left(\hat{W}_{i,j}\left(\lambda\right)\neq 0\right)\,.$$

We aim for this to be approximately equal to some target sparsity level θ. This is done using a fast exponential search algorithm (a generalization of the binary search algorithm for unbounded lists, see Algorithm 2), that exploits the fact that $\mathrm{spar}\left(\lambda\right)$ is non-increasing in λ. This monotonic behavior can be observed from the fixed point of the FISTA algorithm

$$\mathbf{W}=\max\left(1-\frac{\lambda/l}{\left|\mathbf{W}-\frac{1}{l}\nabla f\left(\mathbf{W}\right)\right|},0\right)\left[\mathbf{W}-\frac{1}{l}\nabla f\left(\mathbf{W}\right)\right]\,.$$

Note that as we increase λ, the max operation in the right hand side produces zero for more and more components. Therefore, the number of zeros components is clearly non-decreasing with λ.

Algorithm 2 An exponential search algorithm for setting λ.

Input Target sparsity level - θ, tolerance level - ϵ, measured sparsity - $\mathrm{spar}(\lambda)$.

Initialize Initial point λ, some constant $\rho > 1$, $\lambda_L = -1$, $\lambda_H = -1$.

Repeat:

If $|\mathrm{spar}(\lambda) - \theta| < \epsilon$, **then Return** λ; **Elseif** $\mathrm{spar}(\lambda) < \theta$**, then** $\lambda_H = \lambda$; **Else**, $\lambda_L = \lambda$.

If $\lambda_H = -1$**, then** $\lambda = \rho\lambda$; **Elseif** $\lambda_L = -1$, **then** $\lambda = \lambda/\rho$; **Else**, $\lambda = (\lambda_H + \lambda_L)/2$.

C.3 Lipschitz constant

In the FISTA algorithm (Algorithm 1), we are required to calculate the Lipschitz constant of $\nabla L(\mathbf{W})$. A Lipschitz constant l of a function f is defined through

$$\forall \mathbf{x}, \mathbf{y} : \ \ \|f(\mathbf{x}) - f(\mathbf{y})\| \leq l \|\mathbf{x} - \mathbf{y}\| \ .$$

We obtain that, in our case,

$$l = \frac{\pi}{8} \max_i h(m_i) \lambda_{\max} \left[\mathbf{\Sigma}^{(0)}\right] \tag{49}$$

is the Lipschitz constant, where $\lambda_{\max}[\mathbf{X}]$ is the maximal eigenvalue of $\mathbf{X}$. Begin by observing that for each row of the profile loglikelihood (Eq. 20)

$$\begin{aligned}
& \left\|\nabla_{\mathbf{W}_{i,\cdot}} L(\mathbf{W}) - \nabla_{\mathbf{W}'_{i,\cdot}} L(\mathbf{W}')\right\| \\
= & \left\|\frac{\pi}{8} h(m_i) \frac{\mathbf{W}_{i,\cdot}\mathbf{\Sigma}^{(0)}}{\sqrt{1 + \frac{\pi}{8}\mathbf{W}_{i,\cdot}\mathbf{\Sigma}^{(0)}\mathbf{W}_{i,\cdot}^{\top}}} - \frac{\pi}{8} h(m_i) \frac{\mathbf{W}'_{i,\cdot}\mathbf{\Sigma}^{(0)}}{\sqrt{1 + \frac{\pi}{8}\mathbf{W}'_{i,\cdot}\mathbf{\Sigma}^{(0)}\mathbf{W}'^{\top}_{i,\cdot}}}\right\| \\
\overset{(1)}{\leq} & \left\|-\frac{\pi}{8} h(m_i)\left(\mathbf{W}_{i,\cdot} - \mathbf{W}'_{i,\cdot}\right)\mathbf{\Sigma}^{(0)}\right\| \\
\overset{(2)}{\leq} & \left[\frac{\pi}{8} h(m_i) \lambda_{\max}\left[\mathbf{\Sigma}^{(0)}\right]\right]^2 \left\|\mathbf{W}_{i,\cdot} - \mathbf{W}'_{i,\cdot}\right\| ,
\end{aligned}$$

where we in (1) we used the fact that $\mathbf{\Sigma}^{(0)}$ is positive semi-definite, and in (2) we use the definition of the maximal eigenvalue. If we want a single Lipschitz constant for the all the rows, then we simply maximize over all the rows, and obtain Eq. 49.

D The integral approximation

Recall that

$$f(x) = \frac{1}{1 + e^{-x}}.$$

D.1 Accuracy of the approximation

Our derivation of the simplified profile loglikelihood in section 2.1 used an integral approximation from [23] (Eq. 14), which we write here again for convenience

$$\int_{-\infty}^{\infty} \log(1 + e^x) \mathcal{N}\left(x|\mu, \sigma^2\right) dx \approx \sqrt{1 + \pi\sigma^2/8} \log\left(1 + \exp\left(\frac{\mu}{\sqrt{1 + \pi\sigma^2/8}}\right)\right) . \tag{50}$$

In Fig S2, *left*, We see that the approximation becomes relatively less accurate at low values of μ. Recall that in our derivations μ was the empirical average of the input current to the neuron. Therefore, for low firing rates, the weight estimated using the simplified profile loglikelihood will become inaccurate. This can be partially corrected by adjusting the amplitudes of the weights after the estimation (section D.3). However, in section D.2 we present a more accurate (and less heuristic) approach, which approximates the gradient of the loglikelihood (instead of the likelihood itself). The advantage of this approach is that instead of Eq. 50, we are using

$$\int_{-\infty}^{\infty} f\left(u\right)\mathcal{N}\left(u|\mu,\sigma^2\right)du \approx f\left(\frac{\mu}{\sqrt{1+\frac{\pi}{8}\sigma^2}}\right) \tag{51}$$

which is a more accurate approximation (Fig S2, *right*), that also appears in [23]. To derive this equation, simply differentiate Eq. 50 by μ after changing the integration variable to $t = x - \mu$ (and then change back). Note that [23] also reported better results using the expected gradient (calculated using Eq. 51) instead of the expected loglikelihood (calculated using Eq. 50), but it was not completely clear why is that the case.

For the parameter ranges we tested in our simulations the approximation in Eq. 51 worked quite well. However, if μ is sufficiently low, then even the approximation in Eq. 51 becomes too inaccurate. In that case, one can find more accurate ways to approximate either of these integrals. For example, we can add higher order corrections or divide the integral into regions; calculate the integral numerically (*e.g.*, using Gaussian quadratures or sampling); or save the output of the integral, for different values of μ and σ, in a 2D look-up table. However, such methods typically slow down the algorithm. Another option is to replace the logistic neuron model (Eq. 1) with a more tractable one, such as

$$P\left(S_{i,t}|U_{i,t}\right) = \min\left[1, \exp\left(U_{i,t}\left(2S_{i,t}-1\right)\right)\right],$$

in which the Gaussian integral on the expected loglikelihood (or its gradient) can be calculated exactly.

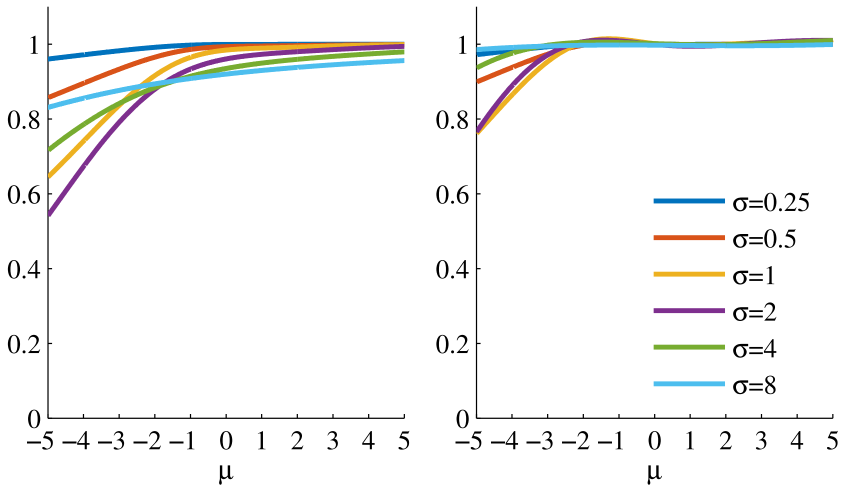

Figure S2. Visualization of the approximation in Eq. 50 (left) and Eq. 51 . For different values of μ and σ, we plot the ratio of the left hand side (calculated by sampling) divided by the right hand side. The approximation on the right is more accurate since the usually ratio is closer to one, and on a larger range.

D.2 Gradient adjustment

Next, we will calculate the gradient of the exact loglikelihood and then approximate the gradient using similar approximations as in section 2.1. In all derivations we will again assume $\mathbf{G}=0$. The end results (derivation in section D.2.1) are

$$\frac{1}{T}\frac{\partial}{\partial b_i}\ln P\left(\mathbf{S}|\mathbf{W},\mathbf{b}\right)\approx m_i-f\left(\frac{\mu_i}{\sqrt{1+\frac{\pi}{8}\sigma_i^2}}\right) \tag{52}$$

$$\frac{1}{T}\frac{\partial}{\partial W_{i,j}}\ln P\left(\mathbf{S}|\mathbf{W},\mathbf{b}\right)\approx \Sigma_{i,j}^{(1)}+m_im_j-m_jf\left(\frac{\mu_{i,(j)}}{\sqrt{1+\frac{\pi}{8}\sigma_{i,(j)}^2}}\right). \tag{53}$$

where we defined

$$\mu_i\triangleq\sum_{k=1}^{N}W_{i,k}m_k+b_i \tag{54}$$

$$\sigma_i^2\triangleq\sum_{r=1}^{N}\sum_{k=1}^{N}W_{i,r}W_{i,k}\Sigma_{k,r}^{(0)} \tag{55}$$

$$\mu_{i,(j)}\triangleq\mu_i+\frac{1}{m_j}\sum_{k=1}^{N}W_{i,k}\Sigma_{k,j}^{(0)} \tag{56}$$

$$\sigma_{i,(j)}^2\triangleq\sigma_i^2-\frac{1}{\Sigma_{j,j}^{(0)}}\left(\sum_{k=1}^{N}W_{i,k}\Sigma_{k,j}^{(0)}\right)^2. \tag{57}$$

A few comments:

1. Note that the bias gradient (Eq. 52) is identical to the bias gradient of the approximate loglikelihood, since if the gradient of the bias is equated to zero, we just obtain Eq. 15 again. However, the gradient of the weights is not equal to same gradient of the approximate loglikelihood.

2. We can plug the maximizer of $\mathbf{b}$ into the gradient of $\mathbf{W}$ (Eq. 53), similarly to what we did with to obtain the profile loglikelihood, but the result does not simplify as nicely as before.

3. Using these expressions we again calculate analytically the adjusted versions of the Hessian (not shown) and the maximum likelihood (section D.2.2).

4. The computational efficiency of calculating the adjusted gradients is $O\left(N^3\right)$, similar to that of the unadjusted gradients.

5. When using these algorithms in the context of the FISTA algorithm (section C.1), we heuristically use the same Lipschitz constant as before, multiplied by 100 (as a "safety margin", since this is only an approximation of the actual constant).

D.2.1 Derivation of adjusted gradient

Recall that, originally,

$$\begin{aligned}
\ln P\left(\mathbf{S}|\mathbf{W},\mathbf{b}\right) &= \sum_{i=1}^{N}\sum_{t=1}^{T}\ln\left[\frac{e^{S_{i,t}U_{i,t}}}{1+e^{U_{i,t}}}\right] \\
&= \sum_{i=1}^{N}\sum_{t=1}^{T}\left[S_{i,t}\left(\sum_{j=1}^{N}W_{i,j}S_{j,t-1}+b_i\right)-\ln\left(1+\exp\left(\sum_{j=1}^{N}W_{i,j}S_{j,t-1}+b_i\right)\right)\right].
\end{aligned}$$

Therefore, the bias gradient (Eq. 52) is

$$\begin{aligned}
&\frac{1}{T}\frac{\partial}{\partial b_i}\ln P\left(\mathbf{S}|\mathbf{W},\mathbf{b}\right) \\
=\;& \frac{1}{T}\sum_{t=1}^{T}\left[S_{i,t}-f\left(U_{i,t}\right)\right] \\
=\;& \left\langle S_{i,t}\right\rangle_T-\left\langle f\left(U_{i,t}\right)\right\rangle_T \\
\overset{(1)}{\approx}\;& m_i-\int f\left(u\right)\mathcal{N}\left(u|\left\langle U_{i,t}\right\rangle_T,\mathrm{Var}_T\left(U_{i,t}\right)\right)du \\
\overset{(2)}{\approx}\;& m_i-f\left(\frac{\mu_i}{\sqrt{1+\frac{\pi}{8}\sigma_i^2}}\right),
\end{aligned}$$

where we used the following:

1. The central limit approximation [24].
2. Eq. 54-55, where $\mu_i=\left\langle U_{i,t}\right\rangle_T$, $\sigma_i^2=\mathrm{Var}_T\left(U_{i,t}\right)$, and the approximation in Eq. 51.

Next, we calculate the weights gradient (Eq. 53)

$$\begin{aligned}
&\frac{1}{T}\frac{\partial}{\partial W_{i,j}}\ln P\left(\mathbf{S}|\mathbf{W},\mathbf{b}\right) \\
=\;& \left\langle S_{i,t}S_{j,t-1}\right\rangle_T-\left\langle f\left(U_{i,t}\right)S_{j,t-1}\right\rangle_T \\
\overset{(1)}{=}\;& \Sigma_{i,j}^{(1)}+m_im_j-\left\langle f\left(U_{i,t}\right)|S_{j,t-1}=1\right\rangle_T\left\langle S_{j,t-1}\right\rangle_T \\
\overset{(2)}{\approx}\;& \Sigma_{i,j}^{(1)}+m_im_j-\left\langle S_{j,t-1}\right\rangle_T\int f\left(u\right)\mathcal{N}\left(u|\left\langle U_{i,t}|S_{j,t-1}=1\right\rangle_T,\mathrm{Var}_T\left(U_{i,t}|S_{j,t-1}=1\right)\right)du \\
\overset{(3)}{\approx}\;& \Sigma_{i,j}^{(1)}+m_im_j-m_jf\left(\frac{\mu_{i,(j)}}{\sqrt{1+\frac{\pi}{8}\sigma_{i,(j)}^2}}\right)
\end{aligned}$$

where we used the following:

1. We defined $\left\langle X_t|Y_t=y\right\rangle_T$ as the conditional empirical mean over X_t given that $Y_t=y$, and used the fact that $\left\langle S_{j,t-1}\right\rangle_T$ is also the empirical frequency of the event $S_{j,t-1}=1$,
2. The central limit theorem [24], and we defined $\mathrm{Var}_T\left(X_t|Y_t=y\right)$ as the conditional empirical variance.

3. The approximation in Eq. 51, and Eqs. 56-57, where we defined

$$\mu_{i,(j)} \triangleq \langle U_{i,t}|S_{j,t-1}=1\rangle_T \;;\; \sigma^2_{i,(j)} \triangleq \mathrm{Var}_T\left(U_{i,t}|S_{j,t-1}=1\right)$$

These last two equations give Eqs. 56-57, since

$$\begin{aligned}
\langle U_{i,t}|S_{j,t-1}=1\rangle_T &= \sum_{k=1}^{N} W_{i,k}\left\langle S_{k,t-1}|S_{j,t-1}=1\right\rangle_T + b_i \\
&= \sum_{k=1}^{N} W_{i,k}\frac{\langle S_{k,t-1}S_{j,t-1}\rangle_T}{\langle S_{j,t-1}\rangle_T} + b_i \\
&= \sum_{k=1}^{N} W_{i,k}\left(m_k + \frac{\Sigma^{(0)}_{k,j}}{m_j}\right) + b_i \\
&= \langle U_{i,t}\rangle_T + \frac{1}{m_j}\sum_{k=1}^{N} W_{i,k}\Sigma^{(0)}_{k,j}
\end{aligned}$$

and, similarly,

$$\begin{aligned}
\mathrm{Var}_T\left(U_{i,t}|S_{j,t-1}=1\right) &= \sum_{k=1}^{N} W_{i,k}\sum_{r=1}^{N} W_{i,r}\mathrm{Cov}_T\left(S_{r,t-1},S_{k,t-1}|S_{j,t-1}=1\right) \\
&\overset{(1)}{\approx} \sum_{k=1}^{N} W_{i,k}\sum_{r=1}^{N} W_{i,r}\left(\Sigma^{(0)}_{r,k} - \Sigma^{(0)}_{r,j}\Sigma^{(0)}_{k,j}/\Sigma^{(0)}_{j,j}\right) \\
&= \mathrm{Var}_T\left(U_{i,t}\right) - \sum_{k=1}^{N} W_{i,k}\sum_{k=1}^{N} W_{i,r}\left(\Sigma^{(0)}_{r,j}\Sigma^{(0)}_{k,j}/\Sigma^{(0)}_{j,j}\right) \\
&= \mathrm{Var}_T\left(U_{i,t}\right) - \left(\sum_{k=1}^{N} W_{i,k}\Sigma^{(0)}_{k,j}\right)^2/\Sigma^{(0)}_{j,j}\,.
\end{aligned}$$

where in (1) we made a purely heuristic approximation in which we calculate the conditional covariance $\mathrm{Cov}_T\left(S_{r,t-1},S_{k,t-1}|S_{j,t-1}=1\right)$ as if $\mathbf{S}$ is Gaussian. As $\mathbf{S}$ is far from Gaussian, this approximation is inaccurate. However, using the accurate result,

$$\mathrm{Cov}_T\left(S_{r,t-1},S_{k,t-1}|S_{j,t-1}=1\right) = \frac{\langle S_{r,t-1},S_{k,t-1}S_{j,t-1}\rangle_T}{\langle S_{j,t-1}\rangle_T} - \frac{\langle S_{k,t-1}S_{j,t-1}\rangle_T}{\langle S_{j,t-1}\rangle_T}\frac{\langle S_{r,t-1}S_{j,t-1}\rangle_T}{\langle S_{j,t-1}\rangle_T} \quad (58)$$

would require the third order moments of the spikes, which will increase computational complexity. Empirically, this seems unnecessary, since in our simulations the performance of the algorithm hardly changes if we simply ignore any dependence of the covariance on $S_{i,t-1}$ and approximate

$$\mathrm{Cov}_T\left(S_{r,t-1},S_{k,t-1}|S_{j,t-1}=1\right) \approx \mathrm{Cov}_T\left(S_{r,t-1},S_{k,t-1}\right).$$

As a middle ground between accuracy and computational complexity, we used the Gaussian approximation. This is a reasonable approximation since the resulting covariance is never negative, and equals zero if $k=j$ or $r=j$, similar to Eq. 58. Note also the expression we used for $\langle S_{k,t-1}|S_{j,t-1}=1\rangle_T$ is the same as the one we would have if used if we assumed that $\mathbf{S}$ was Gaussian. However, it is possible that under some circumstances (*e.g.*, highly synchronous firing modes), the accurate expression (Eq. 58) will be required.

D.2.2 Maximum Likelihood

In this section, we analytically calculate the maximum likelihood solution after the gradient adjustment. The resulting solution (Eq. 73 below, with constants defined in Eqs. 67-69, 71 and 72) is different than the solution before the gradient adjustment (Eq. 25), as expected.

To find the maximum likelihood solution, we require that

$$\frac{\partial}{\partial b_i} \ln P\left(\mathbf{S}|\mathbf{W},\mathbf{b}\right) = 0 \tag{59}$$

and

$$\frac{\partial}{\partial W_{i,j}} \ln P\left(\mathbf{S}|\mathbf{W},\mathbf{b}\right) = 0\,. \tag{60}$$

The solution of Eq. 59, has been calculated already, and is given by Eq. 15

$$b_i = \sqrt{1+\pi\sum_{k,j} W_{i,j}\Sigma^{(0)}_{k,j}W_{i,k}/8}f^{-1}\left(m_i\right) - \sum_{k=1}^{N} W_{i,k}m_k\,, \tag{61}$$

where $f^{-1}\left(x\right) = \ln\left(\frac{x}{1-x}\right)$. Using Eqs. 53-57, the solution of Eq. 60 is given by

$$\begin{aligned} f^{-1}\left(\frac{\Sigma^{(1)}_{i,j}+m_im_j}{m_j}\right) &= \frac{\mu_{i,(j)}}{\sqrt{1+\frac{\pi}{8}\sigma^2_{i,(j)}}} \\ &= \frac{\sum_{k=1}^N W_{i,k}m_k + b_i + \sum_{k=1}^N W_{i,k}\left(\Sigma^{(0)}_{k,j}\left(1-m_j\right)/\Sigma^{(0)}_{j,j}\right)}{\sqrt{1+\frac{\pi}{8}\left(\sum_{r=1}^N\sum_{k=1}^N W_{i,r}W_{i,k}\Sigma^{(0)}_{k,r} - \left(\sum_{k=1}^N W_{i,k}\Sigma^{(0)}_{k,j}\right)^2/\Sigma^{(0)}_{j,j}\right)}} \end{aligned}$$

Substituting 61 into this equation, and denoting

$$c_i \triangleq \left(\mathbf{W}\mathbf{\Sigma}^{(0)}\mathbf{W}^\top\right)_{i,i} = \sum_{k,j} W_{i,j}\Sigma^{(0)}_{k,j}W_{i,k} \tag{62}$$

we obtain

$$f^{-1}\left(\frac{\Sigma^{(1)}_{i,j}+m_im_j}{m_j}\right) = \frac{\sqrt{1+\pi c_i/8}f^{-1}\left(m_i\right) + \sum_{k=1}^N W_{i,k}\left(\Sigma^{(0)}_{k,j}\left(1-m_j\right)/\Sigma^{(0)}_{j,j}\right)}{\sqrt{1+\frac{\pi}{8}\left(c_i - \left(\sum_{k=1}^N W_{i,k}\Sigma^{(0)}_{k,j}\right)^2/\Sigma^{(0)}_{j,j}\right)}}\,. \tag{63}$$

Further denoting

$$A_{i,j} \triangleq \frac{\Sigma^{(0)}_{j,j}}{1-m_j}\left[f^{-1}\left(\frac{\Sigma^{(1)}_{i,j}+m_im_j}{m_j}\right)\sqrt{1+\frac{\pi}{8}\left(c_i - \left(\sum_{k=1}^N W_{i,k}\Sigma^{(0)}_{k,j}\right)^2/\Sigma^{(0)}_{j,j}\right)} - \sqrt{1+c_i\pi/8}f^{-1}\left(m_i\right)\right] \tag{64}$$

Eq. 63 simplifies to

$$A_{i,j} = \sum_{k=1}^N W_{i,k}\Sigma^{(0)}_{k,j},$$

or, in vector notation

$$\mathbf{W} = \mathbf{A}\left(\boldsymbol{\Sigma}^{(0)}\right)^{-1} .$$

Substituting this into the definition of c_i and $A_{i,j}$ we have

$$c_i = \left(\mathbf{A}\left(\boldsymbol{\Sigma}^{(0)}\right)^{-1}\mathbf{A}^\top\right)_{i,i} = \sum_{r=1}^{N}\sum_{k=1}^{N} A_{i,r}A_{i,k}\left[\left(\Sigma^{(0)}\right)^{-1}\right]_{k,r} \tag{65}$$

and

$$A_{i,j} \;\; = \;\; \frac{\Sigma^{(0)}_{j,j}}{1-m_j}\left[f^{-1}\left(\frac{\Sigma^{(1)}_{i,j}+m_i m_j}{m_j}\right)\sqrt{1+\frac{\pi}{8}\left(c_i - A^2_{i,j}/\Sigma^{(0)}_{j,j}\right)} - \sqrt{1+\pi c_i/8}f^{-1}\left(m_i\right)\right] . \tag{66}$$

Shifting terms and squaring Eq. 66, we obtain

$$\begin{aligned} &\left(\frac{1-m_j}{\Sigma^{(0)}_{j,j}}\right)^2 A^2_{i,j} + 2\sqrt{1+\pi c_i/8}f^{-1}\left(m_i\right)\left(\frac{1-m_j}{\Sigma^{(0)}_{j,j}}\right)A_{i,j} + \left(1+\pi c_i/8\right)\left(f^{-1}\left(m_i\right)\right)^2 \\ = \;\; &\left(f^{-1}\left(\frac{\Sigma^{(1)}_{i,j}+m_i m_j}{m_j}\right)\right)^2\left(1+\frac{\pi}{8}\left(c_i - A^2_{i,j}/\Sigma^{(0)}_{j,j}\right)\right) , \end{aligned}$$

which gives the quadratic equation in $A_{i,j}$

$$\begin{aligned} 0 \;\; = \;\; &\left[\left(\frac{1-m_j}{\Sigma^{(0)}_{j,j}}\right)^2 + \frac{\pi}{8\Sigma^{(0)}_{j,j}}\left(f^{-1}\left(\frac{\Sigma^{(1)}_{i,j}+m_i m_j}{m_j}\right)\right)^2\right]A^2_{i,j} + 2\left[\sqrt{1+\pi c_i/8}f^{-1}\left(m_i\right)\left(\frac{1-m_j}{\Sigma^{(0)}_{j,j}}\right)\right]A_{i,j} \\ + \;\; &\left(1+\frac{\pi}{8}c_i\right)\left[\left(f^{-1}\left(m_i\right)\right)^2 - \left(f^{-1}\left(\frac{\Sigma^{(1)}_{i,j}+m_i m_j}{m_j}\right)\right)^2\right] . \end{aligned}$$

Defining

$$F_{i,j} \triangleq \left(1-m_j\right)^2 + \frac{\pi}{8}\Sigma^{(0)}_{j,j}\left(f^{-1}\left(\frac{\Sigma^{(1)}_{i,j}+m_i m_j}{m_j}\right)\right)^2 , \tag{67}$$

and

$$B_{i,j} \triangleq \frac{\Sigma^{(0)}_{j,j}}{F_{i,j}}\left(1-m_j\right)f^{-1}\left(m_i\right) , \tag{68}$$

$$C_{i,j} \triangleq \frac{\left(\Sigma^{(0)}_{j,j}\right)^2}{F_{i,j}}\left[\left(f^{-1}\left(m_i\right)\right)^2 - \left(f^{-1}\left(\frac{\Sigma^{(1)}_{i,j}+m_i m_j}{m_j}\right)\right)^2\right] , \tag{69}$$

the quadratic equation becomes

$$A^2_{i,j} + 2\sqrt{1+\pi c_i/8}B_{i,j}A_{i,j} + \left(1+\frac{\pi}{8}c_i\right)C_{i,j} = 0 ,$$

whose solution is

$$A_{i,j} = \sqrt{1+\pi c_i/8}D_{i,j} , \tag{70}$$

where

$$D_{i,j} \triangleq -B_{i,j} \pm \sqrt{B_{i,j}^2 - C_{i,j}}\,. \tag{71}$$

We chose the solution of the quadratic equation ($\pm$) by requiring that the sign of $D_{i,j}$ be equal to the sign of $\Sigma_{i,j}^{(1)}$ (though this is somewhat heuristic). Substituting this into Eq. 65, we obtain

$$(1+\pi c_i/8)\sum_{r=1}^{N}\sum_{k=1}^{N} D_{i,r}D_{i,k}\left[\left(\Sigma^{(0)}\right)^{-1}\right]_{k,r} = c_i,$$

so

$$c_i = \frac{\sum_{r=1}^{N}\sum_{k=1}^{N} D_{i,r}D_{i,k}\left[\left(\Sigma^{(0)}\right)^{-1}\right]_{k,r}}{1-\frac{\pi}{8}\sum_{r=1}^{N}\sum_{k=1}^{N} D_{i,r}D_{i,k}\left[\left(\Sigma^{(0)}\right)^{-1}\right]_{k,r}}\,.$$

Substituting this into Eq. 70, we obtain

$$A_{i,j} = \frac{D_{i,j}}{\sqrt{1-\frac{\pi}{8}\sum_{r=1}^{N}\sum_{k=1}^{N} D_{i,r}D_{i,k}\left[\left(\Sigma^{(0)}\right)^{-1}\right]_{k,r}}}\,. \tag{72}$$

And therefore, the maximum likelihood estimate of the weights is

$$\mathbf{W} = \mathbf{A}\left(\mathbf{\Sigma}^{(0)}\right)^{-1}\,, \tag{73}$$

where $\mathbf{A}$ can be calculated using Eqs. 67-69, 71 and 72.

D.3 Alternative method - amplitude adjustment

In some cases, we might want to use the original gradient in Eq. 21 , rather than the adjusted gradient. This tends to cause some inaccuracy in our estimates, and mostly in the weight gains and biases. To partially correct for this error, we can re-estimate the gains and biases in the following way. Suppose we obtain a MAP estimate $\hat{\mathbf{W}}$ after using the profile likelihood (Eq. 8). Next, we examine again the original likelihood (without any approximation)

$$\ln P\left(\mathbf{S}|\mathbf{b},\mathbf{W}\right) = \sum_{i=1}^{N}\left[\sum_{t=1}^{T} S_{i,t}\left(\sum_{j=1}^{N} W_{i,j}S_{j-1}+b_j\right) - \sum_{t=1}^{T}\ln\left(1+\exp\left(\sum_{j=1}^{N} W_{i,j}S_{j-1}+b_j\right)\right)\right] + C\,.$$

We assume that the MAP estimate is accurate, up to a scaling constant in each row, so $W_{i,j} = a_i\hat{W}_{i,j}$, and we obtain

$$\begin{aligned}
&\sum_{i=1}^{N}\left[\sum_{t=1}^{T} S_{i,t}\left(a_i\sum_{j=1}^{N}\hat{W}_{i,j}S_{j-1}+b_j\right) - \sum_{t=1}^{T}\ln\left(1+\exp\left(a_i\sum_{j=1}^{N}\hat{W}_{i,j}S_{j,t-1}+b_j\right)\right)\right] + C \\
= \;& T\sum_{i=1}^{N}\left[a_i\sum_{j=1}^{N}\hat{W}_{i,j}\left\langle S_{i,t}S_{j,t-1}\right\rangle_T + b_j\left\langle S_{i,t}\right\rangle_T - \left\langle \ln\left(1+\exp\left(a_i\sum_{j=1}^{N}\hat{W}_{i,j}S_{j,t-1}+b_j\right)\right)\right\rangle_T\right] \quad (74) \\
\approx \;& T\sum_{i=1}^{N}\left[a_i\sum_{j=1}^{N}\hat{W}_{i,j}\left(\tilde{\Sigma}_{i,j}^{(1)}+\tilde{m}_i\tilde{m}_j\right) + b_j\tilde{m}_i - \left\langle \ln\left(1+\exp\left(a_i z_{i,t}+b_j\right)\right)\right\rangle_T\right] + C\,, \quad (75)
\end{aligned}$$

where in the last line we used the expected loglikelihood approximation with CLT again, and denoted (Recall Eqs. 9-10)

$$z_{i,t} \sim N\left(\sum_{j=1}^{N} \hat{W}_{i,j}\tilde{m}_j, \sum_{j,k=1}^{N} \hat{W}_{i,j}\hat{W}_{i,k}\tilde{\Sigma}_{j,k}^{(0)}\right). \quad (76)$$

Sampling $z_{i,t}$ from 76 (we found that about 10^3 samples was usually enough), we can calculate the expectation in (Eq. 74). Next, we can now maximize the likelihood in (Eq. 74) for each gain a_i and bias b_i, separately $\forall i$, by solving an easy 2D unconstrained optimization problem. The new gains can now be used to adjust our estimation of $\mathbf{W}$.

E Alternative inference methods - unobserved spikes as latent variables

Conceptually, having missing spike observations in the data should make it much harder to estimate $\mathbf{W}$ from data. This is because, in this case, we need to modify the posterior (Eq. 4), and replace the complete likelihood $P(\mathbf{S}|\mathbf{W},\mathbf{b})$ with the incomplete likelihood $P(S_{\text{obs}}|\mathbf{W},\mathbf{b})$, where

$$S_{\text{obs}} \triangleq \{\forall i,t : S_{i,t}|O_{i,t}=1\},$$

i.e., the set of all observed spikes from $\mathbf{S}$. However, in order to obtain $P(S_{\text{obs}}|\mathbf{W},\mathbf{b})$ from $P(\mathbf{S}|\mathbf{W},\mathbf{b})$, we need to perform an intractable marginalization over an exponential number of unobserved spike configurations.

The method we derived here sidesteps this problem. We infer the connectivity matrix $\mathbf{W}$ using a MAP estimate, derived using an approximation of the complete loglikelihood $\ln P(\mathbf{S}|\mathbf{W},\mathbf{b})$ (Eq. 4). Though the complete loglikelihood includes all the spikes (even the unobserved ones), in its approximated form we can also handle missing observations. This is done by simply ignoring missing observations and re-normalizing accordingly the sufficient statistics (Eq. 16-17) of the approximated loglikelihood (Eq. 8). This procedure does not affect the asymptotic value of the sufficient statistics, since the observations and spikes are uncorrelated.

In contrast, classical inference methods [25] treat the unobserved spikes as latent variables, and attempt to infer them using one of these standard approaches:

- Markov chain Monte Carlo (MCMC) - which samples the latent variables and weights from the complete posterior.
- Expectation Maximization (EM) - which provides an estimator that locally optimizes the posterior of W given the observed data.
- Variational Bayes (VB) - which approximates the complete posterior using a factorized form.

We experimented with all these approaches on the posterior, without using any of the simplifying approximations used in our main method (section 2). In order to clarify the motivation for our main method, we briefly describe these alternative approaches below, including their advantages and disadvantages.

E.1 Markov chain Monte Carlo (MCMC)

We assume a sparsity promoting spike and slab prior on the weights (here, and everywhere in this section, $\delta(x)$ is Dirac's delta)

$$P_0(\mathbf{W}) = \prod_{i,j}\left[(1-p_0)\,\delta(W_{i,j}) + p_0\mathcal{N}\left(W_{i,j}|\mu_0,\sigma_0^2\right)\right], \quad (77)$$

i.e., each weight is either zero, with probability $1-p_0$, or normally distributed with mean μ_0 and variance σ_0^2, with probability p_0. Note the standard L1 prior on $\mathbf{W}$ (Eq. 46) would not promote sparsity in this MCMC setting, since then every sample of $\mathbf{W}$ would be non-zero with probability 1. We use a Gibbs approach to sample jointly from $P(\mathbf{S},\mathbf{W}|S_{\text{obs}},\mathbf{b})$: first we sample $\mathbf{S}$ given the observed data and the current sample from $\mathbf{W}$ (section E.1.1), and then sample $\mathbf{W}$ given $\mathbf{S}$ (section E.1.2).

In general, computational speed is the main disadvantage of the MCMC method - both for the sampling the spikes and for sampling the weights. For large networks, this approach can only be used if the sampling scheme is completely parallelized over many processors. However, MCMC performs rather well on small networks, similarly to our main method (section 2). An important advantage of the MCMC method over our main method is that it can be used even if some "neuron pairs" are never observed, *i.e.* if for some i,j, $\langle O_{i,t}O_{j,t-k}\rangle_T$ for $k=0$ or 1. Therefore, the MCMC method might be used to complement our main method to infer the weights in this case. It should also be noted that the MCMC approach offers somewhat simpler methods for hyperparameter selection (via sampling from the hyperparameter posterior), and easier incorporation into larger hierarchical models that can include richer prior information about the network and enable proper sharing of information across networks; these are important directions for future research.

E.1.1 Sampling the spikes

In the first step we draw one sample from the posterior of $\mathbf{S}$ given the observed data and an estimated $\mathbf{W}$, using the Metropolized Gibbs sampler [26, 27]. This sampler is quite simple and is highly parallelizable in this setting, because the graphical model representing the posterior $p(\mathbf{S}|S_{\text{obs}},\mathbf{W},\mathbf{b})$ is local: $\mathbf{S}_{\cdot,t}$ is conditionally independent of $\mathbf{S}_{\cdot,t+2}$ given $\mathbf{S}_{\cdot,t+1}$ since the spiking at time t only directly affects the spiking in the next time step (*i.e.*, model 1-2 can be considered a Markov chain in $\mathbf{S}_{\cdot,t}$). Therefore we can alternately sample the spiking vectors $\mathbf{S}_{\cdot,t}$ at all odd times t completely in parallel, and then similarly for the even times. In more general GLM models, where the neuronal input depends on previous k spikes, *i.e.*, $\{\mathbf{S}_{\cdot,t-k}\}_{l=1}^{k}$, the algorithm could be executed using $T/(k+1)$ parallel computations. We note that more sophisticated sampling methods have been developed for this type of problem [28]; these specialized methods are significantly more efficient if implemented serially, but do not parallelize as well as the simple Gibbs-based approach we used here. The performance of the sampler is exemplified in Fig S3, bottom. As can be seen in this figure, usually (unless connectivity weights are very high) the spike sampler can predict the spikes only in a "small neighborhood" of the visible spikes.

First, recall again Eq. 1

$$P\left(S_{i,t}|U_{i,t}\right)=\frac{e^{S_{i,t}U_{i,t}}}{1+e^{U_{i,t}}}\,.$$

and Eq. 2 (with $\mathbf{G}=0$)

$$\mathbf{U}_{\cdot,t}\triangleq\mathbf{W}\mathbf{S}_{\cdot,t-1}+\mathbf{b}\,.$$

We denote here $S_{/(i,t)}$ to be all the component of $\mathbf{S}$ without the $S_{i,t}$ component. In order to do Gibbs sampling, we need to calculate

$$\ln P\left(S_{i,t}|S_{/(i,t)},\mathbf{W},\mathbf{b}\right)=\ln P\left(S_{i,t}|\mathbf{S}_{\cdot,t-1},\mathbf{W},\mathbf{b}\right)+\ln P\left(\mathbf{S}_{\cdot,t+1}|\mathbf{S}_{t},\mathbf{W},\mathbf{b}\right)+C\,,$$

where we can neglect any additive constant that does not depend on $S_{i,t}$. On the right hand side, the first term is

$$\begin{aligned}\ln P\left(S_{i,t}|\mathbf{S}_{\cdot,t-1},\mathbf{W},\mathbf{b}\right) &= \ln\left[\frac{e^{S_{i,t}U_{i,t}}}{1+e^{U_{i,t}}}\right]\\ &= S_{i,t}U_{i,t}+C\,,\\ &= S_{i,t}\left(\sum_{k=1}^{N}W_{i,k}S_{k,t-1}+b_i\right)\,,\end{aligned}$$

while the second term is

$$
\begin{aligned}
& \ln P\left(\mathbf{S}_{\cdot,t+1}|\mathbf{S}_{\cdot,t},\mathbf{W},\mathbf{b}\right) \\
= & \sum_{j}\ln\left[\frac{e^{S_{j,t+1}U_{j,t+1}}}{1+e^{U_{j,t+1}}}\right] \\
= & \sum_{j}\left[S_{j,t+1}U_{j,t+1}-\ln\left(1+e^{U_{j,t+1}}\right)\right] \\
= & \sum_{j}\left[S_{j,t+1}\left(\sum_{k=1}^{N}W_{j,k}S_{k,t}+b_{j}\right)-\ln\left(1+\exp\left(\sum_{k=1}^{N}W_{j,k}S_{k,t}+b_{j}\right)\right)\right]+C \\
= & C+\sum_{j}S_{j,t+1}S_{i,t}W_{j,i} \\
- & \sum_{j}\left[\ln\left(1+\exp\left(\sum_{k\neq i}^{N}W_{j,k}S_{k,t}+b_{j}+W_{j,i}\right)\right)-\ln\left(1+\exp\left(\sum_{k\neq i}^{N}W_{j,k}S_{k,t}+b_{j}\right)\right)\right]S_{i,t}\,.
\end{aligned}
$$

Therefore, we can sample the spikes from

$$
P\left(S_{i,t}|S_{/(i,t)},\mathbf{W},\mathbf{b}\right)\quad\propto\quad\exp\left(\alpha_{i,t}S_{i,t}\right)\,,
$$

where

$$
\begin{aligned}
& \alpha_{i,t}\triangleq b_{i}+\sum_{k=1}^{N}W_{i,k}S_{k,t-1} \\
& +\sum_{j}\left[S_{j,t+1}W_{j,i}-\ln\left(1+\exp\left(\sum_{k\neq i}^{N}W_{j,k}S_{k,t}+b_{j}+W_{j,i}\right)\right)+\ln\left(1+\exp\left(\sum_{k\neq i}^{N}W_{j,k}S_{k,t}+b_{j}\right)\right)\right].
\end{aligned}
\tag{78}
$$

Note that, for a given i, $i_{i,t}$ depends only on spikes from time $t-1$ and $t+1$. Therefore, $S_{i,t}$ samples generated at odd times t are independent from samples $S_{i,t'}$ generated at even times t'. Therefore, we can sample $S_{i,t}$ simultaneously for all odd times t, and then sample simultaneously at all even times t'. Such a simple block-wise Gibbs sampling scheme can be further accelerated by using the Metropolized Gibbs method [26], in which we propose a "flip" of our previous sample. So if $S_{i,t}$ is our previous sample and $S'_{i,t}$ is our new sample, we propose that $S'_{i,t}=1-S_{i,t}$ and then accept this proposal with probability

$$
\min\left(1,\frac{1-P\left(S_{i,t}|S_{/(i,t)},\mathbf{W},\mathbf{b}\right)}{1-P\left(S'_{i,t}|S_{/(i,t)},\mathbf{W},\mathbf{b}\right)}\right)\,.
$$

If the proposal is not accepted, we keep our previous sample $S_{i,t}$.

E.1.2 Sampling the weights

In the second step, we sample $\mathbf{W}$ given $\mathbf{S}$. We first note that, with the spike and slab prior, again the posterior factorizes over i:

$$
P(\mathbf{W}|\mathbf{S},\mathbf{b})=\prod_{i}P(\mathbf{W}_{i,\cdot}|\mathbf{S}_{i,\cdot},\mathbf{b}) \tag{79}
$$

$$
=\prod_{i}\left[\prod_{t}\frac{\exp\left(S_{i,t}\left[\sum_{k=1}^{N}W_{i,k}S_{k,t-1}+b_{i}\right]\right)}{1+\exp\left(\left[\sum_{k=1}^{N}W_{i,k}S_{k,t-1}+b_{i}\right]\right)}\prod_{j}\left[\left(1-p_{0}\right)\delta\left(W_{i,j}\right)+p_{0}\mathcal{N}\left(W_{i,j}|\mu_{0},\sigma_{0}^{2}\right)\right]\right]
$$

Thus, we can sample from each $P(\mathbf{W}_{i,\cdot}|\mathbf{S}_{i,\cdot},\mathbf{b})$ in parallel. To sample from $P(\mathbf{W}_{i,\cdot}|\mathbf{S}_{i,\cdot},\mathbf{b})$, we simply Gibbs sample one element $W_{i,j}$ at a time.

We denote here $W_{/(i,j)}$ to be all the components of $\mathbf{W}$ without the $W_{i,j}$ component, and recall that

$$f(x) = \frac{1}{1+e^{-x}}\,.$$

In order to do Gibbs sampling, we need to calculate

$$\ln P\left(W_{i,j}|\mathbf{S},W_{/(i,j)},\mathbf{b}\right) = \ln P\left(\mathbf{S}|\mathbf{W},\mathbf{b}\right) + \ln P_0\left(W_{i,j}\right) + C\,,$$

where, as before, we can neglect on the right hand side any additive constant that does not depend on $W_{i,j}$. The first term on the right hand side is

$$\begin{aligned}
&\ln P\left(\mathbf{S}|\mathbf{W},\mathbf{b}\right)\\
=\;&\sum_t\left[W_{i,j}S_{i,t}S_{j,t-1} - \ln\left(1+\exp\left(\sum_{k=1}^{N}W_{i,k}S_{k,t-1}+b_i\right)\right)\right]+C\\
=\;&\sum_t\left[W_{i,j}S_{i,t}S_{j,t-1} - \ln\left(1+\exp\left(W_{i,j}S_{j,t-1}\right)\exp\left(\sum_{k\neq j}^{N}W_{i,k}S_{k,t-1}+b_i\right)\right)\right]+C\\
\approx\;&\sum_t\left[W_{i,j}S_{i,t}S_{j,t-1} - \ln\left(1+\left(1+W_{i,j}S_{j,t-1}+\frac{1}{2}W_{i,j}^2S_{j,t-1}\right)\exp\left(\sum_{k\neq j}^{N}W_{i,k}S_{k,t-1}+b_i\right)\right)\right]+C \quad (80)\\
=\;&\sum_t\left[W_{i,j}S_{i,t}S_{j,t-1} - \ln\left(1+f\left(\sum_{k\neq j}^{N}W_{i,k}S_{k,t-1}+b_i\right)\left(W_{i,j}S_{j,t-1}+\frac{1}{2}W_{i,j}^2S_{j,t-1}\right)\right)\right]+C\\
\approx\;&\sum_t\left[W_{i,j}S_{j,t-1}\left[S_{i,t}-f\left(\sum_{k\neq j}^{N}W_{i,k}S_{k,t-1}+b_i\right)\right]\right.\\
-\;&\left.\frac{1}{2}W_{i,j}^2S_{j,t-1}\left[f\left(\sum_{k\neq j}^{N}W_{i,k}S_{k,t-1}+b_i\right)-f^2\left(\sum_{k\neq j}^{N}W_{i,k}S_{k,t-1}+b_i\right)\right]\right]\,,
\end{aligned}$$

where in both approximations, we initially assume that a single weight is typically small, *i.e.* $W_{i,j}\ll 1$. Therefore, denoting

$$\begin{aligned}
\omega_{i,j} &\triangleq \sum_t\left[S_{i,t}-f\left(\sum_{k\neq j}^{N}W_{i,k}S_{k,t-1}+b_i\right)\right]S_{j,t-1}\\
\epsilon_{i,j} &\triangleq \sum_t\left[f\left(\sum_{k\neq j}^{N}W_{i,k}S_{k,t-1}+b_i\right)-f^2\left(\sum_{k\neq j}^{N}W_{i,k}S_{k,t-1}+b_i\right)\right]S_{j,t-1}\,,
\end{aligned}$$

we can write

$$P\left(\mathbf{S}|\mathbf{W},\mathbf{b}\right)\propto\exp\left(W_{i,j}\omega_{i,j}-\frac{1}{2}W_{i,j}^2\epsilon_{i,j}\right)\,.$$

Therefore,

$$P\left(W_{i,j}|\mathbf{S},W_{/(i,j)},\mathbf{b}\right)\propto\exp\left(W_{i,j}\omega_{i,j}-\frac{1}{2}W_{i,j}^2\epsilon_{i,j}\right)P_0\left(W_{i,j}\right)\,.$$

Assuming a spike-and-slab prior

$$P_0\left(W_{i,j}\right) = f\left(-h_0\right)\delta\left(W_{i,j}\right) + f\left(h_0\right)\mathcal{N}\left(W_{i,j}|\mu_0,\sigma_0^2\right)\,, \tag{81}$$

we can use standard Gaussian completion to do spike-and-slab completion, re-normalizing to obtain a proper spike-and-slab distribution. This gives

$$P\left(W_{i,j}|\mathbf{S},W_{/(i,j)},\mathbf{b}\right) = f\left(-h_{i,j}\right)\delta\left(W_{i,j}\right) + f\left(h_{i,j}\right)\mathcal{N}\left(W_{i,j}|\mu_{i,j},\sigma_{i,j}^2\right)\,, \tag{82}$$

with

$$\sigma_{i,j}^2 \triangleq \frac{\sigma_0^2}{1+\epsilon_{i,j}\sigma_0^2} \tag{83}$$

$$\mu_{i,j} \triangleq \frac{\sigma_{i,j}^2}{\sigma_0^2}\left[\mu_0 + \sigma_0^2\omega_{i,j}\right] \tag{84}$$

$$h_{i,j} \triangleq h_0 + \frac{1}{2}\ln\left(\frac{\sigma_{i,j}^2}{\sigma_0^2}\right) + \frac{\mu_{i,j}^2}{2\sigma_{i,j}^2} - \frac{\mu_0^2}{2\sigma_0^2}\,. \tag{85}$$

We can than proceed and sample $\mathbf{W}_{\cdot,j}$ from this spike-and-slab distribution (in Eq. 82) - sampling $W_{i,j}$ simultaneously for all i. Now, since we used an approximation that assumes the weights are weak, so this sampling is not exact. Therefore, even if this approximation is very good, we cannot use direct sampling, or the error will accumulate over time catastrophically. To correct his, we use this approximation as a proposal distribution in a Metropolis Hastings simulation scheme [27]. Alternatively, if we do not want to assume weak weights, then we should replace the Taylor expansion around zero in Eq. 80, with a Taylor expansion around the mode of $W_{i,j}$. Such a Laplace approximation approach, similar to the approach discussed in [29], is slower, yet can be more accurate (the acceptance rate was close to one in simulations), especially when the weights are not necessarily small.

E.2 Expectation Maximization (EM)

The EM approach is similar to the methods discussed in [30,31]. The E step requires the computation of an integral over $p(\mathbf{S}|S_{\text{obs}},\mathbf{W})$. Since this integral is high-dimensional and not analytically tractable, we again resort to MCMC methods to sample the spikes, using the same sampler as before. We found that in most cases it was not necessary to take many samples from the posterior; for large enough network sizes N (and for correspondingly long experimental times T), $\mathbf{S}$ is large enough that a single sample contains enough information to adequately estimate the necessary sufficient statistics in the E step, after an initial burn-in period (30 steps was typically enough). See [32] for further discussion. In the M step we perform maximization over the log-posterior averaged over $p(\mathbf{S}|S_{\text{obs}},\mathbf{W})$, with an L1 prior (Eq. 46). This can be done using FISTA (section C.1), or any other tool for L1-penalized GLM estimation, as in [30,31]. The EM approach is is still rather slow, since we need to sample from the spikes. Moreover, the EM approach typically suffered from shrinkage (a decreased magnitude of the non-zero weights) and exhibited worse performance than the MCMC approach (data not shown).

E.3 Variational Bayes (VB)

Using the standard VB approach [25] and the spike and slab prior on the weights (Eq. 77) we can approximate the posterior $P(\mathbf{S},\mathbf{W}|S_{\text{obs}},\mathbf{b})$ using a fully factorized distribution

$$Q\left(\mathbf{S},\mathbf{W}\right) \triangleq \prod_{i,t} q_{i,t}\left(S_{i,t}\right)\prod_{k,r} q_{k,r}\left(W_{k,r}\right)\,, \tag{86}$$

in which the factors are calculated using

$$\begin{aligned}\ln q_{i,t}\left(S_{i,t}\right) &= \ln \mathbb{E}_{/S_{i,t}} P(\mathbf{S},\mathbf{W}|S_{\text{obs}},\mathbf{b}) \\ \ln q_{k,r}\left(W_{k,r}\right) &= \ln \mathbb{E}_{/W_{k,r}} P(\mathbf{S},\mathbf{W}|S_{\text{obs}},\mathbf{b})\,,\end{aligned}$$

where, for any random variable X, $\mathbb{E}_{/X}$ is an expectation performed using the distribution $Q\left(\mathbf{S},\mathbf{W}|X\right)$. This calculation proceeds almost identically to the derivation of the Gibbs samplers (section E.1), except we need to perform the expectation $\mathbb{E}_{/X}$ over the results. These integrals can be calculated using the CLT and the approximations given in [23, sections 2.4 and 4.1].

The factorized form in Eq. 86 assumes that, approximately, all the weights and all the spikes are independent. With this method, which is somewhat faster than MCMC, the mean firing rates of the neurons could be estimated reasonably well, as is exemplified in Fig S3, bottom. Unfortunately, the weight matrix $\mathbf{W}$ cannot be estimated well if fewer than 30% of the neurons are observed in each timestep. This is in contrast to the other methods, in which the observed fraction can be arbitrarily low. We believe this happens because the VB approximation for the spikes ignores spike correlations - which determine the sufficient statistics in this case (as suggested by Eq. 8). However, it is possible that more sophisticated factorized forms (which do not assume spike independence) will still work.

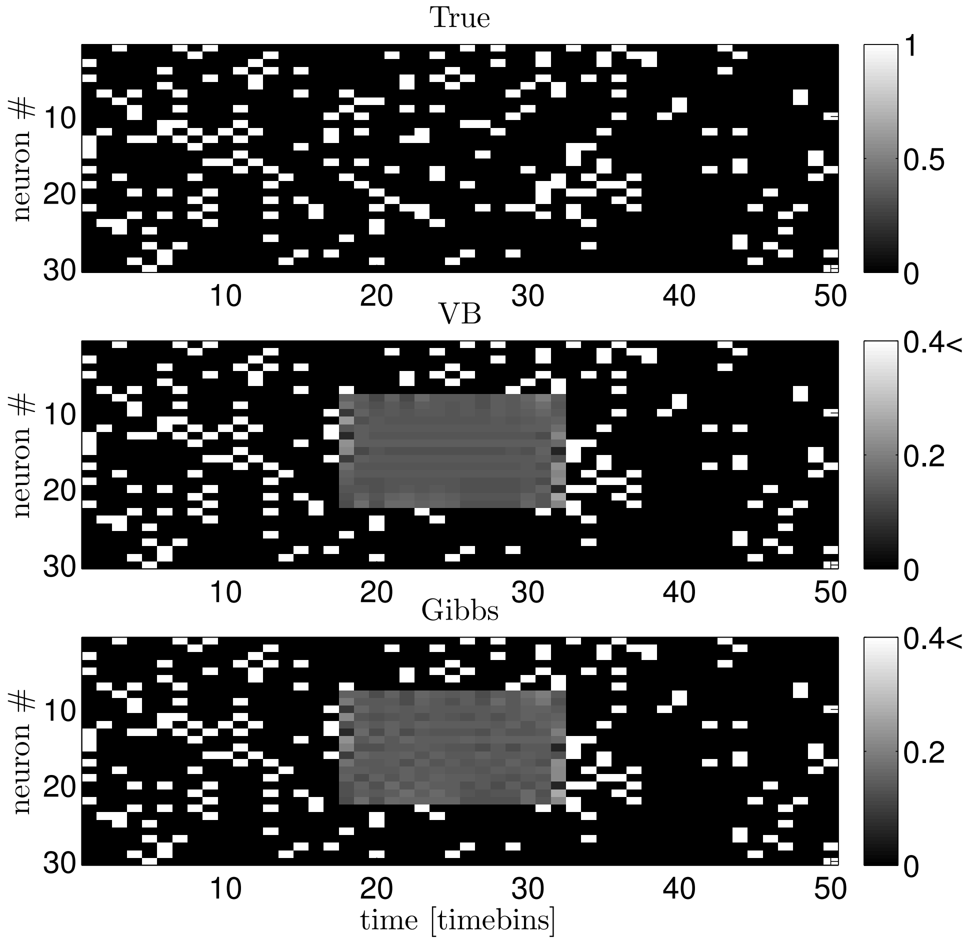

Figure S3. Estimating the spikes using a latent-variable approach with a known network connectivity $(\mathbf{W}, \mathbf{b})$ - visualization. Each raster plot shows the spiking activity in a circular network with local connectivity (spikes are in white). First we show the true spiking activity (*top*). For neurons $10-20$ and times $18-33$ the spiking activity is unobserved. Then, we try to estimate this activity using the Variational Bayes method (VB*, middle*), or using Gibbs sampling (*bottom*). In the unobserved rectangle, the shade indicates the probability of having a spike - between 0 (black) and 1 (white). For visibility, we used saturated shades in the two bottom figures (*i.e.*, values above 0.4 are shown in white). Each neuron in the network has excitatory connections to its neighbours and self-inhibition. Given this connectivity, we can see both methods give reasonable estimates near the edges of unobserved rectangle. Further from the edges, the estimation becomes less certain and converges to the mean spike probability for that neuron.

References

1. Park IM, Pillow JW. Bayesian Spike-Triggered Covariance Analysis. In: Neural Inf Process Syst; 2011. p. 1–9.

2. Ramirez AD, Paninski L. Fast inference in generalized linear models via expected log-likelihoods. J Comput Neurosci. 2014 Apr;36(2):215–234.

3. MacLachlan GJ. Discriminant analysis and statistical pattern recognition. Wiley-Interscience; 1992.

4. Tripathy SJ, Savitskaya J, Burton SD, Urban NN, Gerkin RC. NeuroElectro: a window to the world's neuron electrophysiology data. Front Neuroinform. 2014 Jan;8:40.

5. Braitenberg V, Schüz A. Anatomy of the cortex: Statistics and geometry. Springer-Verlag Berlin Heidelberg; 1991.

6. Cotton RJ, Froudarakis E, Storer P, Saggau P, Tolias AS. Three-dimensional mapping of microcircuit correlation structure. Front Neural Circuits. 2013;7:151.

7. Perin R, Berger TK, Markram H. A synaptic organizing principle for cortical neuronal groups. Proc Natl Acad Sci. 2011;108(13):5419–5424.

8. Robbins H, Monro S. A stochastic approximation method. Ann Math Stat. 1951;22(3):400–407.

9. Ikegaya Y, Sasaki T, Ishikawa D, Honma N, Tao K, Takahashi N, et al. Interpyramid spike transmission stabilizes the sparseness of recurrent network activity. Cereb Cortex. 2012 Feb;23(2):293–304.

10. Song S, Sjöström PJ, Reigl M, Nelson S, Chklovskii DB. Highly nonrandom features of synaptic connectivity in local cortical circuits. PLoS Biol. 2005 Mar;3(3):507–519.

11. Brodal P. The central nervous system: structure and function. Oxford University Press; 2004.

12. van Vreeswijk C, Sompolinsky H. Chaotic balanced state in a model of cortical circuits. Neural Comput. 1998 Aug;10(6):1321–1371.

13. Shu Y, Hasenstaub A, McCormick DA. Turning on and off recurrent balanced cortical activity. Nature. 2003 May;423(6937):288–293.

14. Schneidman E, Berry MJ, Segev R, Bialek W. Weak pairwise correlations imply strongly correlated network states in a neural population. Nature. 2006 Apr;440(7087):1007–1012.

15. Memmesheimer RM, Rubin R, Olveczky BP, Sompolinsky H. Learning precisely timed spikes. Neuron. 2014 May;82(4):925–938.

16. Vidne M, Ahmadian Y, Shlens J, Pillow JW, Kulkarni J, Litke AM, et al. Modeling the impact of common noise inputs on the network activity of retinal ganglion cells. J Comput Neurosci. 2012;33(1):97–121.

17. Pnevmatikakis EA, Gao Y, Soudry D, Pfau D, Lacefield C, Poskanzer K, et al.. A structured matrix factorization framework for large scale calcium imaging data analysis; 2014. http://arxiv.org/abs/1409.2903.

18. Svoboda K. GENIE project; 2015. http://dx.doi.org/10.6080/K02R3PMN.

19. Grewe BF, Langer D, Kasper H, Kampa BM, Helmchen F. High-speed in vivo calcium imaging reveals neuronal network activity with near-millisecond precision. Nat Methods. 2010 May;7(5):399–405.

20. Tibshirani R. Regression shrinkage and selection via the lasso. J R Stat Soc Ser B. 1996;58:267–288.

21. Bach F, Jenatton R, Mairal J, Obozinski G. Convex optimization with sparsity-inducing norms. In: Sra S, Nowozin S, Wright S, editors. Optim Mach Learn. The MIT Press; 2012. p. 19–53.

22. Beck A, Teboulle M. A fast iterative shrinkage-thresholding algorithm for linear inverse problems. SIAM J Imaging Sci. 2009 Jan;2(1):183–202.

23. Wang SI, Manning CD. Fast dropout training. Int Conf Mach Learn. 2013;28:118–126.

24. Diaconis P, Freedman D. Asymptotics of graphical projection pursuit. Ann Stat. 1984;12(3):793–815.

25. Bishop CM. Pattern recognition and machine learning. Singapore: Springer; 2006.

26. Liu JS. Metropolized independent sampling with comparisons to rejection sampling and importance sampling. Stat Comput. 1996 Jun;6(2):113–119.

27. Liu J. Monte carlo strategies in scientific computing. Springer; 2002.

28. Mishchenko Y, Paninski L. Efficient methods for sampling spike trains in networks of coupled neurons. Ann Appl Stat. 2011;5(3):1893–1919.

29. Mohamed S, Heller K, Ghahramani Z. Bayesian and L1 approaches to sparse unsupervised learning. In: Int Conf Mach Learn; 2012. p. 1–8.

30. Pillow JW, Latham P. Neural characterization in partially observed populations of spiking neurons. In: Neural Inf Process Syst; 2007. p. 1–9.

31. Mishchenko Y, Vogelstein JT, Paninski L. A Bayesian approach for inferring neuronal connectivity from calcium fluorescent imaging data. Ann Appl Stat. 2011;5(2B):1229–1261.

32. Diebolt J, Ip E, Olkin I. A stochastic EM algorithm for approximating the maximum likelihood estimate. Stanford University; 1994.
